# Organ-on-a-chip: future of female reproductive pathophysiological models

**DOI:** 10.1186/s12951-024-02651-w

**Published:** 2024-07-31

**Authors:** Zhi-Min Deng, Fang-Fang Dai, Rui-Qi Wang, Hong-Bing Deng, Tai-Lang Yin, Yan-Xiang Cheng, Gan-Tao Chen

**Affiliations:** 1 Department of Obstetrics and Gynecology, Renmin Hospital of Wuhan University, Wuhan, Hubei 430060 China; 2 Reproductive Medicine Center, Department of Obstetrics and Gynecology, Renmin Hospital of Wuhan University, Wuhan, Hubei 430060 China; 3 Department of Gastroenterology, Renmin Hospital of Wuhan University, Wuhan, Hubei 430060 China; 4 Hubei International Scientific and Technological Cooperation Base of Sustainable Resource and Energy, Hubei Key Laboratory of Biomass Resource Chemistry and Environmental Biotechnology, School of Resource and Environmental Science, Wuhan University, Wuhan, Hubei 430060 China

**Keywords:** Organ-on-a-chip, Microfluidics, Microphysiological systems, Uterus-on-a-chip, Endometrial-on-a-chip, Placenta-on-a-chip

## Abstract

The female reproductive system comprises the internal and external genitalia, which communicate through intricate endocrine pathways. Besides secreting hormones that maintain the female secondary sexual characteristics, it also produces follicles and offspring. However, the in vitro systems have been very limited in recapitulating the specific anatomy and pathophysiology of women. Organ-on-a-chip technology, based on microfluidics, can better simulate the cellular microenvironment in vivo, opening a new field for the basic and clinical research of female reproductive system diseases. This technology can not only reconstruct the organ structure but also emulate the organ function as much as possible. The precisely controlled fluidic microenvironment provided by microfluidics vividly mimics the complex endocrine hormone crosstalk among various organs of the female reproductive system, making it a powerful preclinical tool and the future of pathophysiological models of the female reproductive system. Here, we review the research on the application of organ-on-a-chip platforms in the female reproductive systems, focusing on the latest progress in developing models that reproduce the physiological functions or disease features of female reproductive organs and tissues, and highlighting the challenges and future directions in this field.

## Introduction

The female reproductive system comprises the gonads (i.e., ovaries) and the reproductive tract organs (i.e., fallopian tubes, uterus, cervix and vagina), which provide hormonal support and anatomical structures for the reproduction of new offspring [1]. In the past few decades, we have gained a comprehensive understanding of the biological mechanisms underlying the development and physiological functions of the female reproductive systems. This understanding has also advanced the clinical interventions for women’s diseases (such as endometriosis) and reproductive-related diseases (infertility, preterm birth, and abortion, etc.). However, most studies on female reproduction have relied on in vivo animal models and two-dimensional (2D) cell culture models [2]. These models have obvious limitations, as they do not match the complexity and exquisite network architecture of the female reproductive systems [3], nor do they reflect the species-specific differences and dynamic sex hormone levels of women. Moreover, pregnant subjects are usually excluded from clinical trials due to ethical issues in human subject research, which pose a significant challenge to the development of new drugs for the treatment of reproductive and fetal diseases [4]. Therefore, there is an urgent need to develop new in vitro pathophysiological models to study human female reproduction.

The limitations of existing model systems have motivated biomedical engineers to approach female reproductive biology and medicine from an engineering perspective, resulting in a new wave of in vitro models (e.g., biomaterials, three-dimensional (3D) printing, and organs-on-a-chip) [5]. Among them, organ-on-a-chip (OOC) technology attempts to simulate the physiological structure and function of human organs by combining microfluidic technology and tissue engineering technology [6], which has attracted great attention in the biomedical field. Microfluidics is an emerging bioengineering method to control fluids in channels at the micron scale [7], which endows OOC technology with the ability to reconstruct the dynamic flow environment and cell-to-cell interactions in the human body [8], thus providing the possibility of a convenient operation for pharmacokinetic modeling. It can even mimic endocrine signals during the menstrual cycle and pregnancy. OOC models have been applied to recreate the complexity of the human reproductive system and establish a dynamic flow environment of signal factor transport and exchange, which brings new perspectives and hopes to study the molecular biological mechanisms and clinical translational research of specific functions of the female reproductive tract [9].

**Fig. 1:**
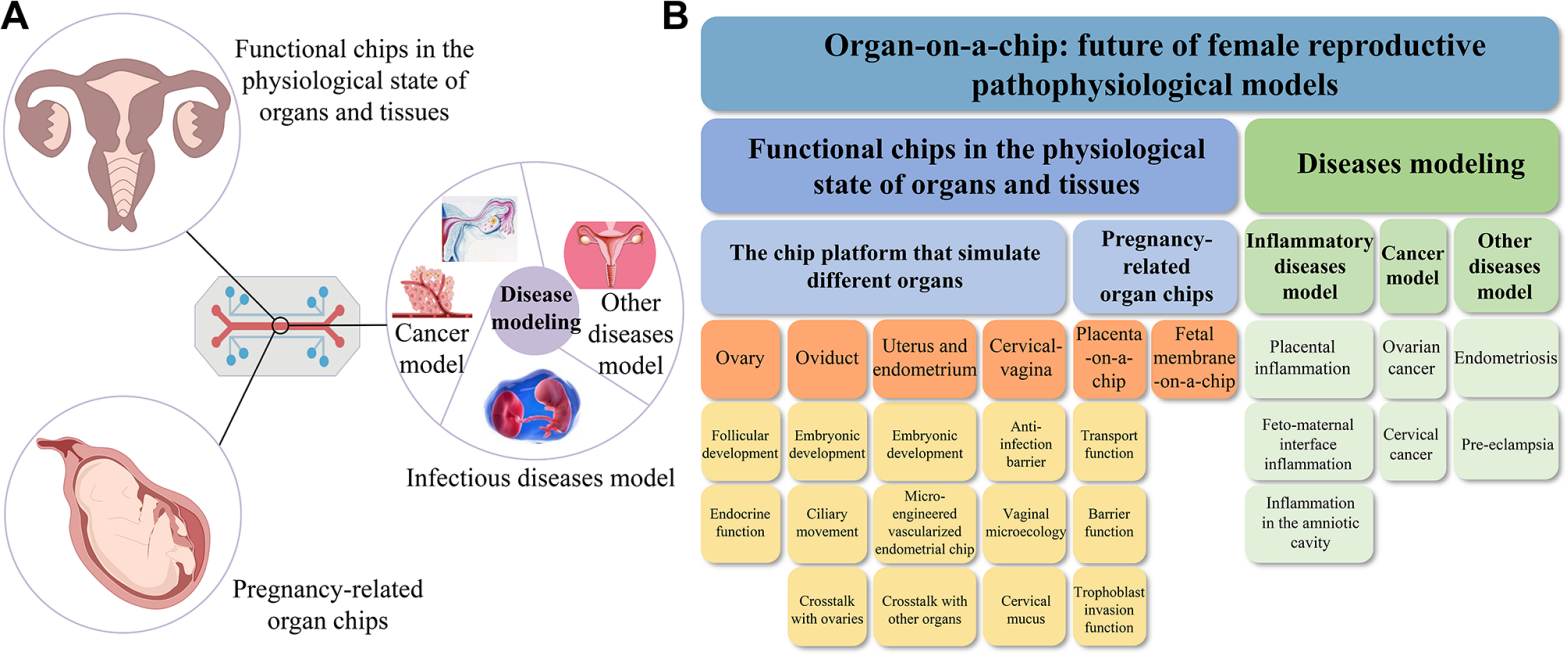
Schematic diagram of this paper

Here, our review aims to review the OOC used to simulate the pathophysiological state of the human female reproductive system (Fig. 1). Firstly, we present the representative development of organ-on-chip technology in this area of research to date, followed by highlighting the advantages of organ-on-chips over other culture models. Then, we summarize the cell types, cell culture methods, engineering materials and techniques used in the construction of the models, and focus on elucidating their ability and potential to simulate the function and physiological state of organs and tissues, including: (i) the functions of various organs and tissues of the female reproductive system and the reproductive science field; (ii) the application in studying the basis and treatment of female reproductive system diseases. Finally, we discuss the future challenges and trends in this field.

## The brief history of OOC for diseases of the female reproductive system

**Fig. 2:**
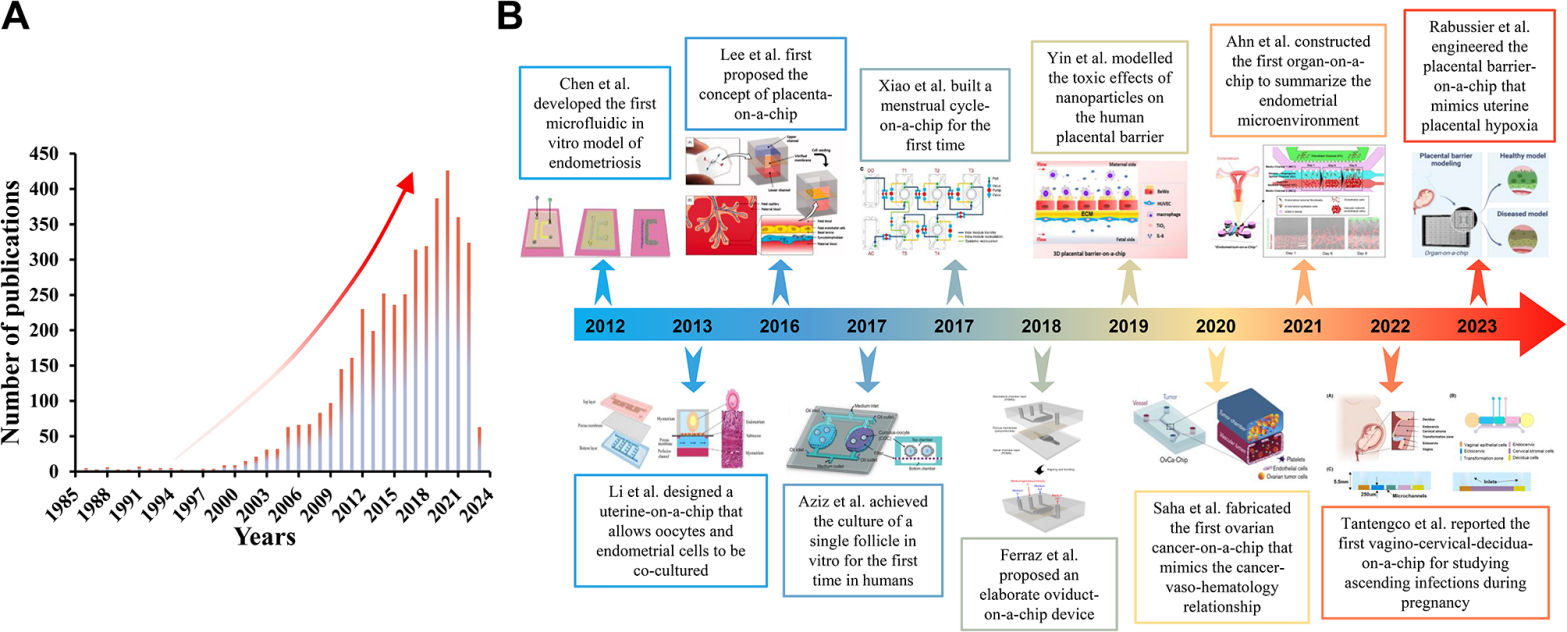
(**A**) Trends in OOC publications related to the female reproductive system in recent years. (Data from Web of Science, using the keywords “chip” and “female reproductive system” from 1985 to 2023). (**B**) Representative advances in OOC applications for the female reproductive system to date

The reproduction of the pathophysiological function of the female reproductive system has gone through a long process, from 2D culture to 3D in vitro culture platform, and gradually evolved from cell level to organ level. With the advancement of technology and demand, OOC has gradually emerged due to its superior ability to recapitulate homeostasis and disease state and has been widely used in the field of biomedical engineering, shining light and heat in the research of multiple human systems [10–12]. The number of OOC related articles published in the field of the female reproductive system has been increasing year by year (Fig. 2A), reaching its peak in recent years. Here, we briefly review the history and development of OOC devices relevant to the study of the female reproductive systems.

*Ingber’s* team first used the term “organ-on-a-chip” in 2010, when they created a biomimetic microsystem of the alveolar-capillary interface on the chip, which reproduced the human lung’s response to bacteria and inflammatory cytokines, to provide low-cost alternatives to animal and clinical studies for drug screening and toxicology applications [13]. In the female reproductive system, in 2012, *Chen et al.* co-cultured endometrial stromal cells (ESCs) and human peritoneal mesothelial cells (HPMCs) on a microfluidic chip to mimic the pathophysiology of peritoneal endometriosis [14]. And then the first OOC of the uterus [15], placenta [16], menstrual cycle [17], fallopian tube [18], and ovarian cancer [19] were developed one after another (Fig. 2B). OOC research on the pathophysiological model of the female reproductive system is more fragmented and complicated than that of other human systems. We collected as much relevant information as possible and organized the research information according to different organs and different research teams. In addition, it is well known that the cell type involved in the in vitro culture model and the medium/microenvironment used will affect the experimental results, so it is important to understand these possible effects when designing experiments. However, we noticed that this part of the content has hardly been summarized in previous reviews in this field. Therefore, we focused on extracting this information and summarizing them in the table during the literature review.

## Advantages of organoids over traditional models

2D cell culture is currently the most prevalent method of cell biology research, which has the advantages of easy operation, low cost of media and materials, relatively simple culture equipment, and suitability for large-scale culture and functional studies [3]. However, cells in 2D culture are grown on flat hard surfaces and usually present a flattened shape, which does not mimic the growth of cells in 3D environments in vivo, and may result in a large discrepancy between cellular behavior and in vivo [20]. Many of the cell biological behavioral data obtained through 2D culture environments are not well reproduced in the corresponding animal models, and drug screening based on 2D cultured cells often encounters inconsistency between in vivo and in vitro efficacy [21].

The limitations of 2D cell culture in mimicking the physiological environment and cell morphology in vivo have prompted researchers to turn to 3D cell culture, which is a type of cell culture that mimics 3D growth environments in vivo [22] by allowing cells to spontaneously aggregate into 3D spheres (e.g., suspension droplet cultures and low-adherence planar cultures, etc.) [23], or using porous membrane co-culture systems (e.g. transwell plates) [24] or allowing cells to adhere, stretch and grow on 3D structures (natural or synthetic cellular scaffolds) with compositional structures similar to solid tissues [25], to co-regulate cellular proliferation and differentiation temporally and spatially. Although 3D culture more realistically reproduces the behavioral properties of cells in an organism compared to 2D cell culture models [3], 3D cell culture has limitations in controllability and reproducibility, and parameters such as cell scaffold structure, porosity, biocompatibility, and cell density limit its application in large-scale and high-throughput experiments [26].

Animal models have become an important tool in the study of human diseases because they can mimic some of the characteristics of human diseases and thus provide valuable information for disease mechanisms, diagnosis and treatment. However, animal models have several limitations. On the one hand, animal models cannot fully represent the physiological, pathological and genetic characteristics of humans, and there are significant species differences between the two, which may lead to the failure of animal models to accurately reflect the onset, progression and response of human diseases [27]. On the other hand, there are some technical (e.g., genetic engineering, chemical induction, exogenous transplantation, etc.) and ethical (e.g., animal welfare, numbers, handling, etc.) challenges in the establishment and use of animal models [28]. These may limit the availability, feasibility and efficiency of animal models.

In view of the above-mentioned limitations of cell culture (2D or 3D) and animal models in simulating tissue interfaces and organ functions, microphysiological system (MPS) has been proposed as an in vitro technique to simulate the pathophysiology of human organs or tissues [29]. The development and application of MPS has attracted global attention, especially in the fields of drug discovery and toxicity screening. The U.S. Food and Drug Administration (FDA) has begun to employ MPS for organ-specific or multi-organ toxicity screening of drugs, food additives, chemicals, cosmetics, and other compounds [30]. This advancement signifies the importance of microphysiological systems in the field of drug development and toxicity screening. However, the applications of microphysiological systems do not stop here. Scientists have further developed OOC technology, a subset of microphysiological systems, which is a microfluidic, miniaturized and flexible device that allows the creation of a functional unit of an organ in vitro using living cells and an organ-specific dynamic microenvironment [8]. OOC technology creates controlled tissue microenvironments by mimicking physiological environments and establishes the functional connectivity of different tissues, providing the necessary support to achieve organ function [31]. OOC technology has the following advantages over traditional models:

i) Closer to the human physiological environment: OOC technology can create a functional unit of an organ in vitro by using living cells and organ-specific dynamic microenvironment, simulating the interaction of cell-cell, cell-extracellular matrix, cell-liquid flow, cell-mechanical force and many other factors. It can also achieve precise control of the cellular microenvironment, such as fluid flow, shear force, oxygen gradient, etc., so as to better reflect the complex dynamic internal environment inside the human body and provide a model basis for understanding the internal mechanism of human biology.
ii) Higher controllability: OOC technology not only enables dynamic stimulation and intervention of cells (e.g., drugs, toxins, pathogens, etc.), but also allows real-time monitoring and analysis of cells (e.g., electrophysiology, fluorescence, drug metabolism, etc.).
iii) Lower time cost and ethical constraints: OOC technology can take advantage of miniaturization, integration and automation to achieve high-throughput, high-efficiency and high-sensitivity drug screening and testing, thus reducing the use and consumption of experimental equipment and animals, and lowering the ethical and legal issues associated with animal experiments.

Overall, the advantages of OOC technology over traditional models (both cellular and animal) lie in its high degree of fidelity, accuracy, controllability and standardization as well as its relatively low cost, resulting in a wider range of applications, which will undoubtedly revolutionize future drug development and biomedical pathophysiological modelling research.

## The ability and potential of OOC to simulate organ tissue function and physiological state

### Organ tissue function and OOC

The female reproductive system consists of the internal and external genitalia, the vulva and its structures form the external genitalia, and the internal genitalia includes a three-part tubular system: fallopian tube, uterus, and vagina [1]. This tubular system is connected to the ovaries, the main reproductive organ. The main functions of the female reproductive system are to produce oocytes, support fetal development, and secrete sex hormones to regulate reproduction. These functions involve complex regulation of endocrine signals within and between organs. Below, we will discuss the progress of OOC technology in simulating the pathophysiology of each organ in female internal genitalia and their interdependent functional connections.

#### Ovary (Table 1, rows 1–6)

**Table 1:** Organ chips that mimic each organ of the female reproductive system

Model category	Cell types	Culture environment	Device characteristics	Culture characteristics	Significance	Year/Reference
Ovary-on-a-chip	λ Early secondary preantral follicles of female deer mice	λ Medium: conditioned medium + 5 µg/ml insulin + 5 µg/ml transferrin + 5 ng/ml selenium + 100 mIU/mL FSH λ Temperature: 37 °C; CO_2_: 5%; Humidity: 95%	λ The first layer: 100 μm λ The additional layer: 50 μm λ The third layer: 50 μm λ Device material: PDMS λ Fabrication method: soft lithography	λ Flow rate: Core: 50 µl/h; Dispatching: 30 µl/h; Shell: 120 µl/h; Oil: 2 ml/h; Aqueous extracting fluids: 4 ml/h. λ Coating: alginate (harder) and collagen (softer)	To reveal the crucial role of mechanical heterogeneity in the mammal ovary in regulating follicle development and ovulation.	2014/ [42]
Ovary-on-a-chip	λ Single human pre-antral follicle	λ Medium: 50% MEM-α + 50% F-12 + 1 mg/mL FBS + 5 µg/mL insulin + 5 µg/mL transferrin human + 5 µg/mL sodium selenite + 3 mg/mL BSA + 1:100 rFSH λ Temperature: 37 °C; CO_2_: 5%; Humidity: 95%	λ Upper PDMS layer: 2 mm λ Middle PDMS layer: 5 mm λ Lower PDMS layer: 1 mm λ Device material: two plates of PMMA substrate + three layers of PDMS plates λ The entire device: 6 × 4 × 0.8 cm^3^ λ Fabrication method: soft lithography	λ Flow rate: 8.33 µL/h λ Coating: 0.5% of sodium alginate and 0.5% of sodium alginate	To culture a single human ovarian follicle and explore the hormonal changes and their interactions during folliculogenesis.	2017/ [43]
Ovary-on-a-chip	λ Ditto	λ Ditto	λ Ditto	λ Ditto	To explore the toxicity and possible mechanisms of doxorubicin	2020/ [44]
Ovary-on-a-chip in mice models	λ Cumulus-oocyte complex of CD-1 female mouse	λ Medium: MEM-α + 10% FBS + 10 ng/mL EGF + cumulus cell expansion stimulator + 25 × 10^− 3^ M HEPES. λ Temperature: 37 °C; O_2_: 95%; CO_2_: 5%	λ Top layer: polyester membrane: 200 μm; acrylic layer: 0.862 mm λ Bottom layer: 200 μm λ Device material: two PDMS layers λ Fabrication method: soft lithography	λ Flow rate: varies from step to step λ Coating: /	For analyzing and screening the effects of potential contraceptive agents on the maturation of the cumulus-oocyte complex.	2019/ [45]
Ovary-on-a-chip in large mammal models	λ Ovarian cortex and follicle of domestic cat and dog	λ Medium: Cat: MEM + 4.2 µg/ml insulin + 3.8 µg/ml transferrin + 5 ng/ml selenium + 2 mM L-glutamine + 100 µg/ml penicillin G sodium and streptomycin sulfate + 0.05 mM ascorbic acid + 0.1% w/v polyvinyl alcohol + 10 ng/ml FSH + 100 ng/ml EGF; Dog: MEM-α + 3 mg/ml BSA + 4.2 µg/ml insulin + 3.8 µg/ml transferrin + 5 ng/ml selenium + 2 mM glutamine + 10 IU/ml penicillin G + 10 µg/ml streptomycin + 10 µg/ml FSH λ Temperature: 38.5 °C; CO_2_: 5%; Humidity:/	λ Top layer: 3 mm PMMA λ Channel layer: 1.5 mm PMMA λ Base layer: 2 mm Polystyrene λ Complete chip: 40 × 24 × 6.5 mm^3^ λ Device material: PMMA and Polystyrene λ Fabrication method: soft lithography	λ Flow rate: 2 µL/min or 10 µL/min λ Coating: 1% alginate hydrogel	To support the in vitro survival of domestic cat and dog follicles enclosed within the ovarian cortex or isolated from the ovarian cortex.	2018/ [46]
Menstrual cycle-on-a- chip/Ovary-on-a-chip/ Multi organ chip	λ Primary/early secondary follicles of female CD-1 mice; Quintet-MFP: murine ovary, human fallopian tube, endometrium, ectocervix, liver tissues	λ Medium: Follicular phase: growth medium (50% MEM-α + 50% F-12 + 3 mg/mL BSA + 0.5 mg/mL bovine fetuin + 5 µg/mL insulin + 5 µg/mL transferrin + 5 µg/mL selenium) + 10 mIU/ml recombinant FSH; Luteal phase: growth media without FSH λ Temperature: 37 °C; CO_2_: 5%	λ Solo-MFP: a coupled donor/acceptor module and a module for tissues λ Duet-MFP: a donor module, two modules for tissues, and a separate acceptor module λ Quintet-MFP: a donor module, five modules for tissues, and a separate acceptor module λ Device material:/ λ Fabrication method: Solo-MFP and Duet-MFP: pneumatic actuation technology; Quintet-MFP: embedded electromagnetic actuation technology	λ Flow rate: Solo-MFP: 40 µL/h; Duet-MFP: 0 µL/h; Quintet-MFP: 100 µL/h λ Coating: 0.5% alginate drop or 1% alginate hydrogels	To develop platforms that could sustain tissue-level function for the length of the human menstrual cycle (that is, 28 days).	2017/ [17]
Oviduct-on-a-chip	λ Bovine oviduct epithelial cells	λ Medium: DMEM/Ham’s F-12 + 5 µg/mL insulin + 5 µg/mL transferrin + 10 mM glutathione + 100 µg/mL gentamycin + 10 ng/mL EGF + 50 nM trans-retinoic acid + 5% FCS + 2.5 mg/mL amphotericin B λ Temperature: 38.5 °C; O_2_: 7%; CO_2_: 5%	λ Two independent compartments: 3 × 2.8 × 0.37mm^3^ λ Device material: PDMS λ Fabrication method: /	λ Flow rate: 5 µL/h λ Coating: /	To investigate the mechanisms related to genetic reprogramming and the degree to which they differ between in vitro and in vivo embryos.	2018/ [18]
Oviduct-on-a-chip	λ Human fallopian tube epithelium	λ Medium: normal: low testosterone concentration of 0.8 nM; PCOS-like: high testosterone concentration of 2 nM; MEM + 0.3% BSA + 0.5 mg/ml fetuin + 1% penicillin/streptomycin + 1% ITS λ Temperature: 37 °C; CO_2_: 5%	λ Solo-MFP: a coupled donor/acceptor module and a module for tissues λ Device material: / λ Fabrication method: Solo-MFP: pneumatic actuation technology	λ Flow rate: 35 ~ 50 µL/h; λ Coating: 0.5% alginate drop or 1% alginate hydrogels	To investigate how exposure to testosterone-rich environments affects the function and gene expression of the human fallopian tube epithelium.	2020/ [54]
Oviduct-on-a-chip	λ Mouse primary oviduct epithelial cells λ Embryos of CD-1 mice	λ Medium: DMEM/F-12 containing 20% FBS λ Temperature: 37 °C; CO_2_: 5% CO_2_ of mouse oviduct secretory epithelial cells while 6% CO_2_ of embryos	λ The circular hole on both ends of the channels: 3 mm λ The chamber: 5 mm λ The whole channels: 20 × 1 × 10mm^3^ λ Device material: PDMS λ Fabrication method: Multilayer soft lithography technology	λ Flow rate: 1.0 µL/h λ Coating: /	Reduce intracellular ROS levels to optimize embryo culture conditions.	2022/ [53]
Uterus-on-a-chip	λ Mouse oocytes λ Mouse endometrial cells	λ Medium: Endometrial cells: M16 medium; Oocytes: M6 medium λ Temperature: 37 °C; CO_2_: 5%	λ Top layer: contained a zigzag shaped channel (500 μm in width and 110 μm in height) λ Porous membrane: pore size of 8 μm λ Bottom layer: contained 4 parallel rectangular channels (6 × 3 × 0.11mm^3^) λ Device material: Top layer and bottom layer: PDMS; Porous membrane: polycarbonate λ Fabrication method: soft lithography technology	λ Flow rate: 10 µL/h λ Coating: 0.5% gelatin solution	To achieve higher morula rates and blastocyte rates.	2013/ [15]
Uterus-on-a-chip	λ Human endometrial stromal cell λ Mouse embryos	λ Medium: Human endometrial stromal cell:75% DMEM and 25% MCDB 105 medium + 100U/ml penicillin + 100U/ml streptomycin + 5 µg/mL insulin + 10% charcoal-stripped FBS + 10% FBS + antibody; Mouse embryos: HTF medium λ Temperature: 37 °C; O_2_: 95%; CO_2_: 5%	λ Concentration gradient generator: width: 250 μm, height: 230 μm λ Diamond-shaped passive micro-mixer: width: 200 μm, height: 230 μm λ Culture chamber: radius: 3 mm, height: 230 μm λ Perfusion channel: width: 250 μm, height: 230 μm λ Device material: PDMS λ Fabrication method: soft lithography technologyλ	λ Flow rate: 1 µL/min λ Coating: /	Successfully demonstrated its ability to support embryonic development from the 8-cell stage to the hatching stage in 48 h.	2016/ [61]
Endometrial perivascular stroma-on-a-chip	λ Human endometrial stromal cells λ HUVECs	λ Medium: HUVECs: EBM-2 medium + EGM™-2 Single Quot growth factors; Human endometrial stromal cells: phenol red-free DMEM/F-12 + 10% charcoal-stripped calf serum + 1nM 17-β estradiol + 1× antibiotic-antimycotic solution (stromal complete growth medium) λ Temperature: 37 °C; O_2_: 95%; CO_2_: 5%; Humidity: saturated humidity	λ Two microfluidic chambers: 4.75 × 6.2mm^2^ λ Porous membrane: pore size of 2 μm λ Device material: PDMS λ Fabrication method: soft lithography technology	λ Flow rate: 2.5µL/min λ Coating: /	The endometrial perivascular stroma model was sustainable for up to 4 weeks.	2017/ [63]
Endometrial perivascular stroma-on-a-chip	λ Human endometrial stromal cells λ HUVECs	λ Medium: HUVECs: EGM™-2MV BulletKit™; Human endometrial stromal cells: phenol red-free DMEM/F-12 + 5% charcoal-stripped calf serum + 1nM 17-β estradiol + 1× antibiotic-antimycotic solution (stromal complete growth medium) λ Temperature: 37 °C; O_2_: 95%; CO_2_: 5%; Humidity: saturated humidity	λ Ditto	λ Flow rate: 1µL/min λ Coating: collagen type IV (10 µg/cm^2^)	Revealed that the perfused-vascular endothelium enhancing the decidualization response.	2019/ [64]
Endometrium-on-a-chip	λ HUVECs λ Human endometrial epithelial cells λ Human endometrial stromal fibroblasts	λ Medium: HUVECs: EGM-2 medium; Endometrial epithelial cells and endometrial stromal fibroblasts: DMEM/F12 + 10% FBS + 1% penicillin–streptomycin λ Temperature: 37 °C; CO_2_: 5%	λ Five microchannels: two central channels (channel SC and channel VC) + fibroblast channel + media channels 1 + media channels 2 λ Device material: PDMS λ Fabrication method: soft lithography and replica molding	λ Flow rate: / λ Coating: fibrin gel solution (2.5 mg/ml fibrinogen with 0.15 U/ml aprotinin)	Recapitulates in vivo endometrial vasculo-angiogenesis and hormonal responses displaying key features of the proliferative and secretory phases of the menstrual cycle.	2021/ [65]
Endometrium-on-a-chip	λ Human endometrial cellular components λ Human ovarian follicular cells λ HUVEC λ Primary human stromal cells	λ Medium: Human endometrial cellular components: StemPro MSC SFM CTS^™^; HUVEC: EBM-2 medium; Primary human stromal cells: DMEM medium + 10%FBS; Human ovarian follicular cells: M-199/MCDB-105(1:1 mixture) + 10%FBS λ Temperature: 37 °C; O_2_: 95%; CO_2_: 5%	λ Dual chamber chip platform: 34 × 34 × 5mm^3^ λ Triangular uterine endometrial chamber: 20 × 16 × 3mm^3^ λ Dual circular ovarian chambers: 7 × 7 × 3mm^3^ λ Media channel: 2 × 3 × 3mm^3^ λ Device material: PDMS λ Fabrication method: 3D printing	λ Flow rate: / λ Coating: /	Developed a ‘dual reproductive organ-on-a-chip’ that was used to predict the reproductive toxicity of various hazardous materials.	2020/ [68]
Cervical epithelial layer-on-a-chip	λ Immortalized ectocervical cells λ Immortalized endocervical epithelial cells	λ Medium: Immortalized ectocervical and endocervical epithelial cells: KSFM medium + 30 µg/mL bovine pituitary extract + 0.1 ng/mL EGF + 0.4 mM CaCl_2_ + 0.5 mg/mL primocin λ Temperature: 37 °C; CO_2_: 5%	λ The first microchannel layer: 5 μm deep λ The second cell culture chamber layer: 500 μm deep λ Device material: PDMS λ Fabrication method: soft lithography technology	λ Flow rate: / λ Coating: IV collagen	To simulate the effects of cell death, migration, EMT, and inflammatory cytokine secretion in bacterial infection and inflammatory states.	2021/ [73]
Vagina-cervix-decidua-organ-on-a-chip	λ Immortalized ectocervical cells λ Immortalized endocervical epithelial cells λ Cervical stromal cells λ Vaginal epithelial cell λ Human decidua cells λ Transformation zone	λ Medium: Vaginal epithelial cells: KSFM medium + 30 µg/mL bovine pituitary extract + 0.1 ng/mL EGF + 0.4 mM CaCl_2_ + 0.5 mg/mL primocin + KGM™-2 Keratinocyte Growth Medium Bulletkit™; Ectocervical epithelial and endocervical epithelial cells: complete KSFM medium; Cervical stromal cells: DMEM/F-12 + 10% FBS + 50 IU/ml penicillin/50 µg/ml streptomycin + 2.5 µg/ml amphotericin B; Human decidual cells: DMEM/F-12 + 10% FBS + 50 IU/ml penicillin/50 µg/ml streptomycin + 2.5 µg/ml amphotericin B λ Temperature: 37 °C; CO_2_: 5%	λ The vagina, ectocervical, transformation zone, cervical stroma, and decidua layers: interconnected by an array of 24 microchannels (300 × 30 × 5µm^3^) λ Endocervical epithelia layer: interconnected by an array of 72 microchannels (600 × 30 × 5µm^3^) λ Device material: PDMS λ Fabrication method: soft lithography technology	λ Flow rate: / λ Coating: IV collagen	*Ureaplasma parvum* infection was found not to promote a large-scale inflammatory response.	2022/ [74]
Vagina-cervix-decidua-organ-on-a-chip	λ Ditto	λ Ditto λ THP-1 monocytes: RPMI 1640 medium + 0.05 mM 2-mercaptoethanol + 10% FBS λ Temperature: 37 °C; CO_2_: 5%	λ Ditto	λ Ditto	Revealed that exosomes from *Ureaplasma parvum*-infected ectocervical epithelial cells promote feto-maternal interface inflammation but are insufficient to cause preterm delivery.	2022/ [75]
Vagina-on-a-chip	λ Human vaginal epithelium cells λ Human vaginal stromal fibroblasts	λ Medium: vaginal epithelium cells: vaginal epithelium growth medium; human uterine fibroblasts cells: fibroblast growth medium (Uterine fibroblasts were used to replace primary human vaginal fibroblasts) λ Temperature: 37 °C; CO_2_: 5%	λ The apical channel: 16.7 × 1 × 1mm^3^ λ The basal channel: 16.7 × 1 × 0.2mm^3^ λ The porous membrane: pore size of 7 μm λ Device material: PDMS λ Fabrication method: /	λ Flow rate: 40µL/h λ Coating: the apical channel: collagen IV (30 µg/mL) and collagen I (200 µg/mL); the basal channel: collagen I (200 µg/mL)	Demonstrates the vagina-on-a-chip can be used to better understand interactions between the vaginal microbiome and host tissues.	2022/ [77]

**Notes**: /: no report; MEM, modified eagle’s medium; FSH, follicle stimulating hormone; FBS, fetal bovine serum; rFSH, recombinant follicle stimulating hormone; PMMA, polymethyl methacrylate; PDMS, polydimethylsiloxane; EGF, epidermal growth factor; BSA, bovine serum albumin; DMEM, Dulbecco’s modified Eagle’s medium; HTF, human tubal fluid; HUVECs, Human Umbilical Vein Endothelial Cells; EGM-2, endothelial growth medium 2; SC, Stroma-Angiogenic Sprout Channel; VC, Vascular Network Channel; EMT, Epithelial–mesenchymal transition; KSFM, keratinocyte serum-free medium

**Fig. 3:**
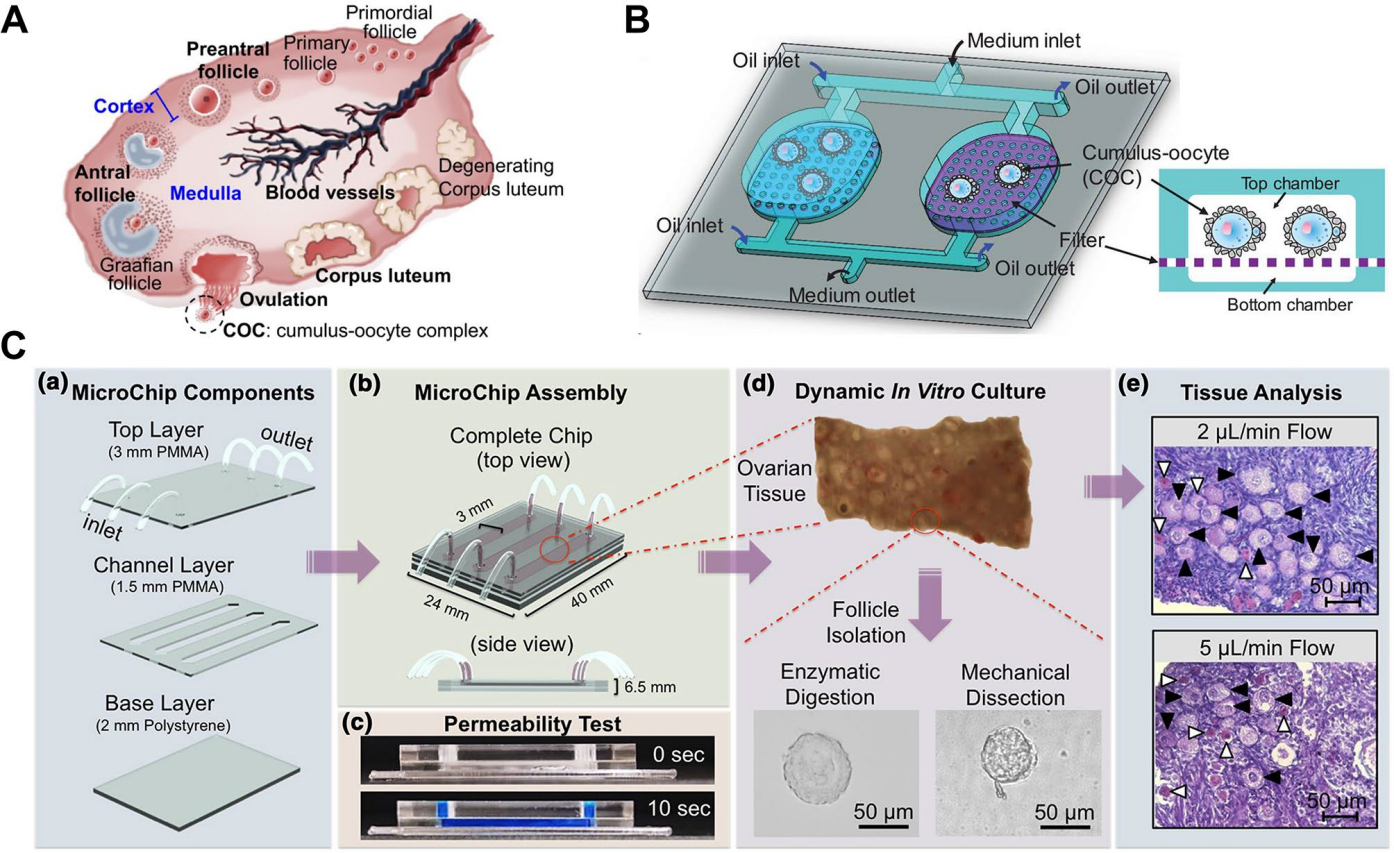
(**A**) Schematic diagram of ovarian follicle development. The outer layer of the ovary is the cortex, the inner layer is the medulla, and the follicles are located in the softer ovarian cortex. During ovulation, the dominant follicle bursts and releases a secondary oocyte, while the other follicles undergo atresia. Reproduced with permission [42]. Copyright 2014 Elsevier Ltd. (**B**) Schematic diagram of a microfluidic microarray for the ovarian follicle-oocyte complex. Reproduced with permission [45]. Copyright 2019, John Wiley and Sons, Ltd. (**C**) Schematic diagram of a microfluidic dynamic in vitro culture system for ovarian follicles in cats and dogs. Reproduced with permission [46]. Copyright 2017 John Wiley & Sons, Ltd

The ovaries are two almond-shaped structures located on both sides of the uterus and connected to the fallopian tubes. The ovaries, as the female gonads, have two main roles. The first is the reproductive function of the ovaries. When a girl enters puberty, each ovary contains thousands of follicles, each of which contains a primary oocyte. As the follicle matures, some primary oocytes become secondary oocytes. By the time of ovulation, only one mature follicle remains and the others degenerate [32] (Fig. 3A). During ovulation (about once a month), the dominant follicle ruptures and releases a secondary oocytes, which can enter the fallopian tube and meet the sperm, and be fertilized to become a zygote [33]. The second is the endocrine function of the ovary, which secretes sex hormones, such as estrogen, progesterone, androgens, and other hormones and growth factors, that nourish nine major systems of human bone, immune, reproductive, nervous and so on, and maintains the menstrual cycle of women [34].

OOC platforms have been widely developed for various organ systems, such as liver [35], lung [36], kidney [37], cardiovascular [38], and gastrointestinal [39]. However, we found that current research on mimicking the development of active oocytes still heavily relies on mouse models, with only a few studies aiming at creating ovaries-on-a-chip. Since there are scarce studies on establishing OOC directly from human ovarian tissue, we comprehensively reviewed the most significant literature in this field, not only limiting the scope to human tissue sources. This paper mainly summarizes two aspects: first, the reproductive function of the ovary, namely, the dynamic chip culture of follicle development in vitro; and second, the endocrine function of the ovary, namely, the chip simulation of the female menstrual cycle.

##### Dynamic chip culture of follicular development in vitro (table 1, rows 1–5)

The mammalian ovary consists of a peripheral cortex and a central medulla, and the follicle is located in the softer ovarian cortex, containing an oocyte and many small follicular cells around it [40]. The number of follicles in a woman’s lifetime is determined before birth and cannot be regenerated afterwards. Therefore, any diseases, drugs, environmental exposures, or factors that can impair the quantity and quality of follicles and/or oocytes, such as genetics, autoimmune, iatrogenic interventions (surgery, radiation therapy, or chemotherapy drugs), and environmental exposures, will increase the risk of premature ovarian insufficiency, hormonal imbalances, and infertility in women [41]. In vitro culture of ovarian tissue or follicles can provide a valuable pathophysiological model for female reproductive science and has significant potential for fertility preservation.

*Choi et al.* encapsulated early secondary antral follicles from Peromyscus in microcapsules composed of a softer, biodegradable collagen (0.5%) hydrogel core and a stiffer, slowly degradable alginate (2%) hydrogel shell layer to mimic ovarian microtissues with mechanical heterogeneity. They used a non-planar microfluidic flow device to provide the ovarian microtissues with a dynamic in vitro culture environment, to revealed the crucial role of mechanical heterogeneity in regulating follicular development and ovulation in mammals [42]. *Aziz et al.* achieved the first in vitro human culture of individual follicles by encapsulating them in calcium alginate hydrogel on a microfluidic chip [43]. They also used the chip to investigate the toxicity of Adriamycin on rat ovarian follicles and possible molecular mechanisms [44]. *Hui et al.*. developed a mouse cumulus-oocyte complex microfluidic chip for analyzing and screening the effects of potential contraceptives on the maturation of cumulus-oocyte complexes [45] (Fig. 3B). In addition to small mammal ovarian tissue, microfluidic culture has been applied to support in vitro survival of pre-antral isolated follicles in domesticated cats and dogs [46] (Fig. 3C).

These microfluidic microarray culture systems for dynamic in vitro follicular development consist of in vitro ovarian microtissues and microfluidic platforms, which simulate follicles in real ovarian tissues by encapsulating follicles of different species from different stages (pre-antral follicles, antral follicles, and cumulus-oocyte complexes) in different carriers, including core-shell microencapsulation of different stiffness, alginate hydrogels, and ovarian cortical tissues. Such in vitro follicular development organoids will enhance our understanding of the mechanisms of follicular formation and be used to investigate the effects of various factors on follicular development and ovulation.

##### Female menstrual cycle organ chips (Table 1, row 6)

The female reproductive system involves complex spatiotemporal patterns of endocrine signals within and between organs, which together dynamically coordinate the development and transport of oocytes, leading to embryo implantation in case of fertilization, or otherwise transition to the menstrual period [47, 48]. In addition to the advantages of integrated miniaturization and automation, an important feature of the microfluidic OOC system is its high-throughput dynamic flow environment, which can precisely control the liquid flow form [49]. Based on this, the researchers developed a microfluidic system that supports mouse ovarian follicles to produce a human 28-day menstrual cycle hormone profile that regulates human female reproductive tract and peripheral tissue dynamics in single, dual, and multiunit microfluidic platforms (called Solo-MFP, Duet-MFP, and Quintet-MPF, respectively) [17]. Among them, the Quintet-MPF system contains five primary cells (human cervix, human fallopian tube, mouse ovary, human uterus, and human liver), which together form a multiorgan chip platform called “EVATAR”. By administering gonadotropins in vitro, ovarian tissue can provide steroids and peptide hormones to downstream fallopian tubes, endometrium, external cervix, and liver tissue, with great potential for drug development (contraception or infertility treatment) and toxicological research [17].

There is a scarcity of OOC studies on the endocrine function of the ovary, and more research is urgently needed. Future research in this field will evolve towards greater accuracy and conformity to physiological fluctuation curves. In addition, promoting vascularization, simulating mechanical strength and dynamic physiology of the ovary, and building a multiorgan chip platform in the ovarian pathophysiology chip model will be a very promising strategy for patients with hypofertility.

#### Fallopian tubes (Table 1, rows 7–9)

**Fig. 4:**
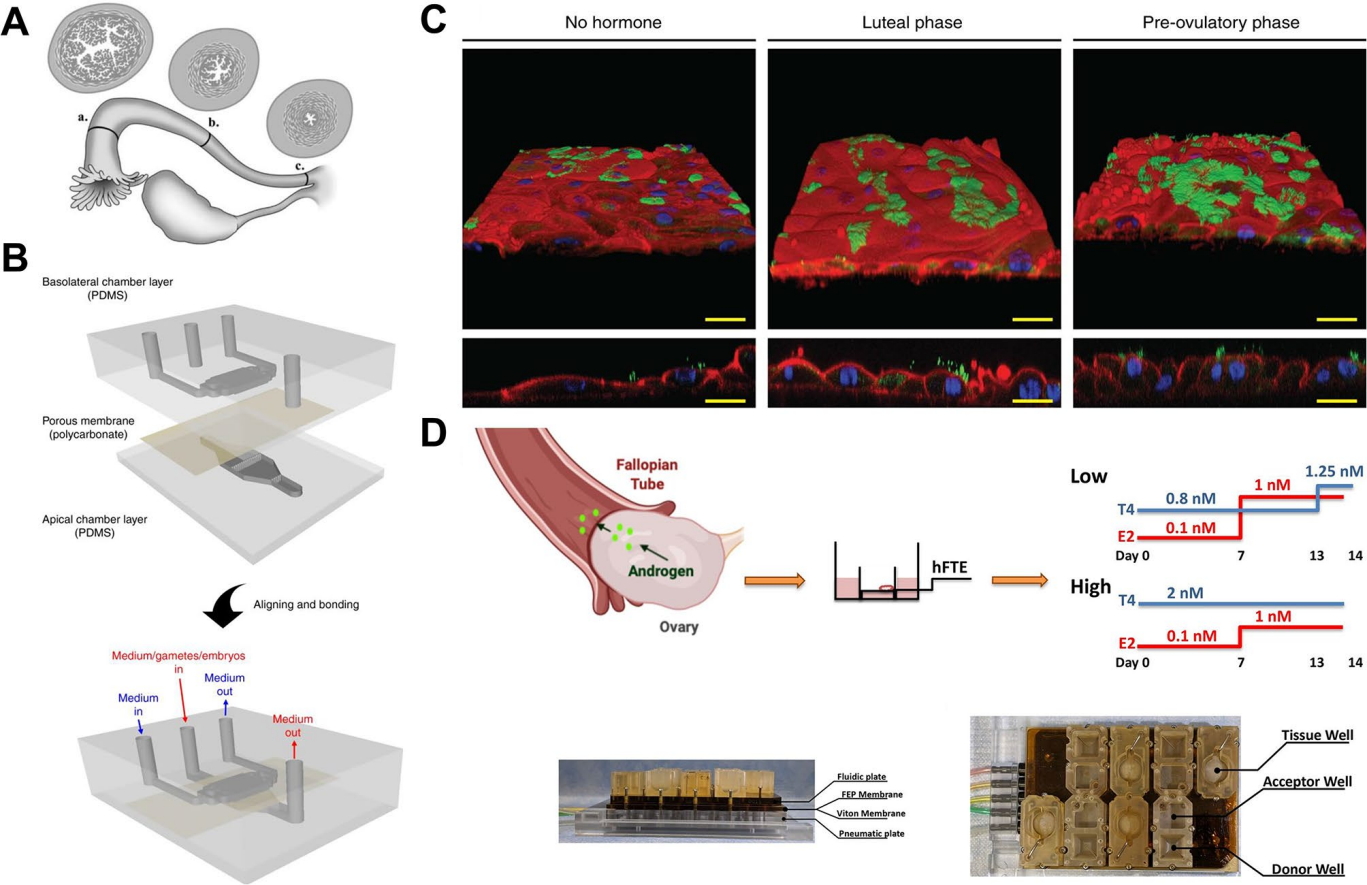
Mimicking the function of the fallopian tube on the chip. (**A**) Illustration of the human Fallopian tube, showing the longitudinal folds in cross-section at the (a) infundibulum, (b) ampulla and (c) isthmus. Reproduced with permission [50]. Copyright 2006 Oxford University Press. (**B**) The device features a complete separation of the apical and basolateral compartments, which allows distinct collection of secreted factors from, or introduction of exogenous factors to, the apical (luminal) and basolateral (blood circulation) compartments. Reproduced with permission [18]. (**C**) Effects of steroid hormone stimulation on bovine oviduct epithelial cells (BOEC) cell and cilia growth and differentiation during the luteal phase and preovulatory phase were simulated using the device in Fig. 4B. 3D reconstruction of confocal immunofluorescent images for cilia (acetylated alpha-tubulin, green), nuclei (HOECHST 33,342, blue), and actin filaments (phalloidin, red). Reproduced with permission [18]. (**D**) Hormonal perfusion of cultured human fallopian tube epithelial (hFTE) tissue with low (0.8 nM) versus high (2 nM) androgen conditions using a pneumatic pump to mimic polycystic ovary syndrome (PCOS) androgen exposure. Reproduced with permission [54]. Copyright 2020 Oxford University Press

The fallopian tube, which connects the ovary and the uterus, has a wall composed of an outer serous layer, a middle muscular layer, and an inner mucosal layer that extends to the endometrium (Fig. 4A). The fallopian tube provides the space and biological environment for embryo development from fertilized oocytes to morula [50, 51]. *Ferraz et al.* reported the first 3D printed device of a semi-tubular porous filter for bovine oviduct epithelial cells (BOEC) culture. The tubal system enabled real-time imaging and supported in vitro fertilization [52], but it did not allow perfusion during embryo culture. A year later, their team developed a more refined oviduct microarray device that features a complete separation of the apical and basolateral compartments, allowing distinct collection of secreted factors from, or introduction of exogenous factors to, the apical (luminal) and basolateral (blood circulation) compartments (Fig. 4B). The device mimicked changes in the estrous cycle and BOEC responded to steroid hormone mimicry during the luteal and preovulatory phases, with cellular and ciliary growth and differentiation under perfusion [18] (Fig. 4C). Both the culture conditions and the estrous cycle changes created a more in vivo-like environment for embryo development. The device also has real-time imaging capabilities that could be used to assess the effects of drugs on gametes or embryos in real time. *Wang et al.* established a fallopian tube-on-a-chip model using mouse primary fallopian tube epithelial cells, and optimized embryo culture conditions by generating more medium displacement through a microfluidic device, thereby reducing ambient reactive oxygen species levels [53].

Besides investigating the function of the fallopian tubes in supporting fertilization and embryo development, microfluidic devices were also applied to study the transport function of human fallopian tube epithelium (hFTE) [54] (Fig. 4D). The inner surface of the fallopian tube consists mainly of epithelial cells with hormone-regulated active cilia, which coordinate with the rhythmic contraction of the fallopian tube smooth muscle, and guide the transport of the embryo towards the uterus [55]. *Tia et al.* isolated fallopian tube epithelium and exposed it to low (0.8 nM) versus high (2 nM) androgen conditions, and then collected tissue samples for imaging to quantify ciliary beating frequency [54]. They found that elevated levels of testosterone altered the expression of several genes that regulate cilia in epithelial cells and negatively affected the cilia beating rate, expanding the application of microfluidic devices in the field of low fertility in women caused by polycystic ovary syndrome and other hyperandrogenism disorders.

A previous study by *Xiao et al.* on the ovary-on-a-chip confirmed the crosstalk between ovaries and fallopian tubes, and showed that the fallopian tube system could respond in real time to estrogen signals from the connected ovarian model [17]. It was also previously reported that ovaries and fallopian tubes communicated through co-culture of hFTE and mouse ovarian follicles, and that the tubal ciliary pulsation and the secretion of oviduct-specific glycoprotein 1 were regulated by dynamic estradiol [56]. These studies confirm that tubal dynamics were closely synchronized with the ovarian cycle, but no further OOC studies are currently available.

Moreover, a microfluidic chip device that maintains stable hormone concentration gradients and tracks individual sperm over long periods of time has been designed, simulating the tubal microenvironment in vivo, which has been used to study the chemotaxis of sperm [57], develop novel sperm chemical inducers [58], and explore the tangled process of sperm escape before fertilization [59]. As this type of research is more focused on the male reproductive system, it is not discussed in detail here. Similarly, *Leemans et al.* used transwell inserts and microfluidic platforms to co-culture differentiated horse oviduct epithelial cells for in vitro dilatation and fertilization, which were beyond the scope of our study [60].

Since artificial reproductive technologies enable complete fertilization and early embryogenesis in vitro without fallopian tubes, the physiology of the human fallopian tubes has been increasingly overlooked, which may account for the scarcity of research on oviduct-on-a-chip. However, as far as in vivo fertilization is concerned, fallopian tubes are essential for reproduction. For the future development of oviduct-on-a-chip, the challenges of simulating the change of tubal diameter, the secretory function of the tube epithelium, the movement of cilia, and the crosstalk between the tube and ovary are undoubtedly to be addressed.

#### Uterus and endometrium (Table 1, rows 10–15)

The uterus is located in the pelvic cavity between the bladder and rectum, and can be divided into the corpus uterus and cervix uterus, with the upper part connecting to the fallopian tube and the lower part of the cervix leading to the vagina. The uterus is a hollow organ with thick muscle walls, consisting of three layers: the outer layer is the serous membrane, the middle layer is the smooth muscle layer, and the inner layer is the endometrium. The endometrium dynamically sheds and regenerates in each menstrual cycle in response to ovarian steroid hormones. This multilayered organ comprises several different cell types, including lumen and glandular epithelial cells, endometrial stromal cells (ESCs), immune cells, and blood vessel cells that form spiral arteries.

In this section of the literature review, we summarize the different research focuses on uterus-on-a-chip, which can be categorized into three aspects: (i) uterus-on-a-chip that simulates the process of fertilization and embryonic development; (ii) micro-engineered vascularized endometrial chip that mimics the endometrial vascular system; and (iii) multi-organ chips that model the endocrine interactions between the uterus and other organs. We elaborate on these aspects in the following paragraphs.

##### Uterus-on-a-chip for the study of the fertilization process and embryo development

**Fig. 5:**
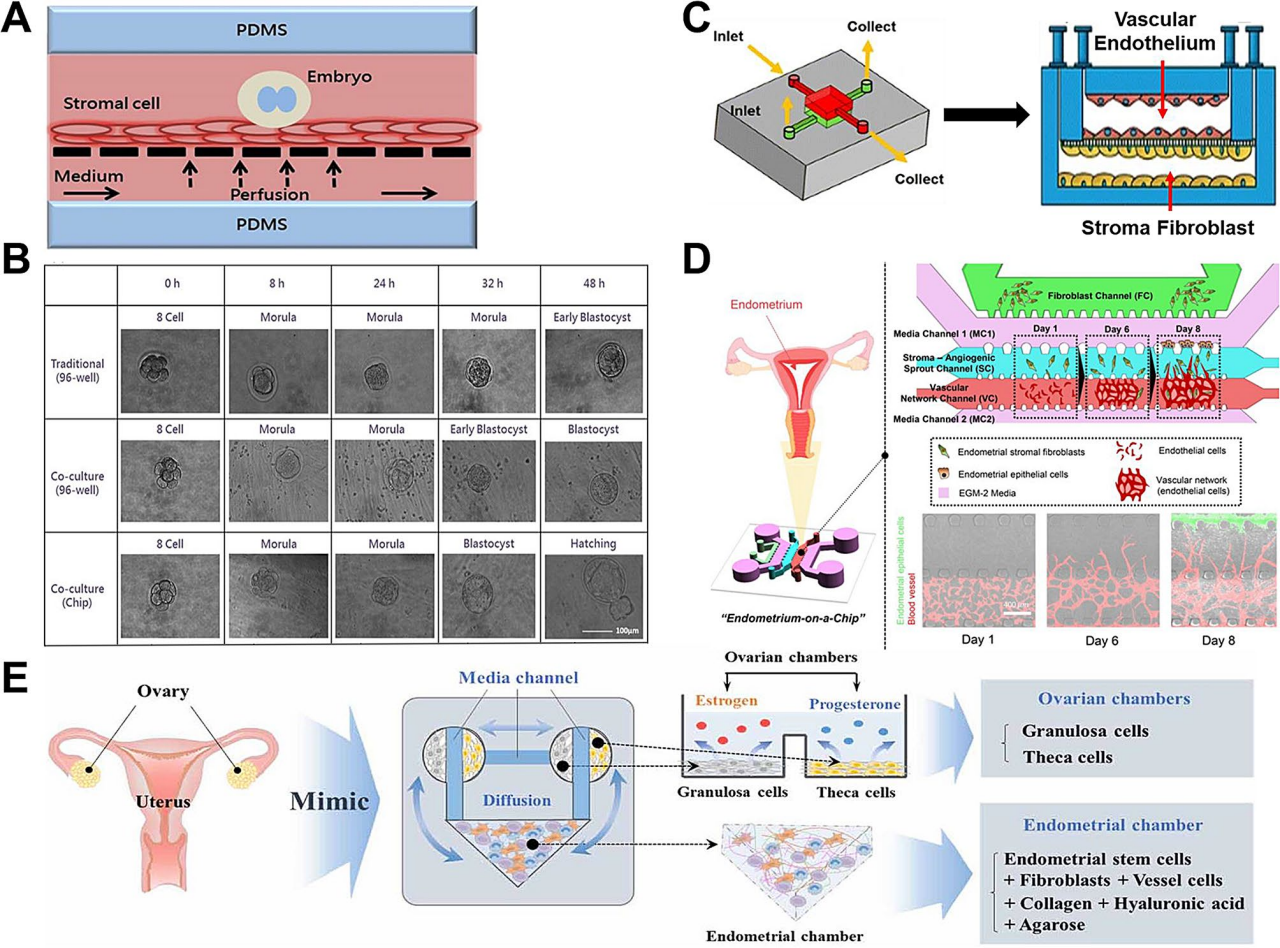
Mimicking the uterus and endometrium on the chip. (**A**) A uterine-on-a-chip co-cultured with oocytes and endometrial stromal cells. Perfusion channels at the bottom of the uterine bionic microfluidic chip provide luteinizing hormone and estrogen through the porous membrane pores to the endometrial stromal cells cultured on the porous membrane, which regulate the proliferation and differentiation of the endometrial tissues to facilitate successful embryo implantation. Reproduced with permission [15]. Copyright 2015 Elsevier B.V. (**B**) Fertilized embryos cultured in the microfluidic uterine microarrays of Fig. 5A showed a significant increase in the rate of embryo development compared to culturing in 96-well plates alone or co-culturing with endometrial stromal cells in 96-well plates. Reproduced with permission [15]. Copyright 2015 Elsevier B.V. (**C**) Perivascular endometrial stromal microarrays composed of human primary umbilical vein endothelial cells and endometrial stromal cells. Reproduced with permission [63]. Copyright 2019 Oxford University Press. (**D**) Micro-engineered vascularized endometrial chips consisting of human endometrial epithelial cells, stromal fibroblasts and vascular endothelial cells. Angiogenic activity increased significantly with culture time. Reproduced with permission [65]. Copyright 2021 Oxford University Press. (**E**) Dual reproductive organ chip to simulate cross-talk between ovary and endometrium. The ovarian chamber contains granulosa cells and theca cells while the endometrial chamber contains endometrial stem cells, fibroblasts, vessel cells, collagen, hyaluronic acid and agarose. Reproduced with permission [68]

In 2013, *Li et al.* developed a microfluidic uterus-on-a-chip that enabled co-culture of oocytes and endometrial cells and implemented procedures for fertilization and embryonic development. They found that the uterus-on-a-chip achieved higher morula and blastocyst rates than the static culture model [15]. The embryonic and endometrial cells used in the study were derived from mice. Later, *Chang et al.* attempted to isolate ESCs from human body and co-culture them with mouse embryos in order to more closely mimic human physiology. The perfusion channel at the bottom of this uterus-mimicking microfluidic chip provided progesterone and estrogen through the porous membrane pores to the ESCs cultured on the porous membrane, which were used to regulate the proliferation and differentiation of the endometrial tissues in order to facilitate successful embryo implantation (Fig. 5A). Fertilized embryos cultured in this microfluidic uterus chip showed a significant increase in the rate of embryo development compared to culturing in 96-well plates alone and co-culturing with endometrial stromal cells in 96-well plates [61] (Fig. 5B).

The limitation of such type of models is that the oocytes or fertilized embryos used are mostly of murine origin due to ethical constraints. Furthermore, compared with the real uterus, this kind of uterus-on-a-chip device lacks complex cellular components and often have only a single endometrial stromal cell.

##### Micro-engineered vascularized endometrial chip for studying the endometrial vascular system

The endometrium undergoes decidualization in the late secretory stage of pregnancy under the continuous influence of progesterone, which provides nourishment and immune tolerance for embryo implantation and placental development. Decidualization is a morphological and physiological transformation of endometrial stromal cells to adapt to pregnancy [62]. Decidualization of maternal tissue is a crucial initial step in pregnancy, and researchers have attempted to apply the organ-on-a-chip approach to simulate this female reproductive process. For instance, *Gnecco et al.* co-cultured human umbilical vein endothelial cells (HUVECs) and ESCs to mimic the hormonal changes during the idealized 28-day menstrual cycle and evaluated the decidualization capacity of stromal cells by measuring prolactin production and cell morphological changes. Notably, the co-culture system enabled simultaneous analysis of decidualization of uterine stromal cells, remodeling of vascular endothelial cells, and vascular barrier formation. Moreover, the endometrial perivascular stromal model was sustainable for up to 4 weeks, which was sensitive to hormones and suitable for quantitative biochemical analysis [63] (Fig. 5C). Their team further refined the chip model in some details, finding that stromal decidualization was significantly enhanced when endothelial cells were exposed to hemodynamic forces (such as laminar shear stress) from controlled microfluidic perfusion and demonstrated that hemodynamic forces modulate decidualization [64]. *Ahn et al.* further integrated three cell types, human endometrial epithelial cells, stromal fibroblasts and vascular endothelial cells, to construct a micro-engineered vascularized endometrial microarray, demonstrating that angiogenesis is critical during the menstrual cycle and embryo implantation [65] (Fig. 5D). We can observe that more and more cell types are being incorporated in the study of endometrial vasculature with the advancement of engineered vascular and OOC technology.

##### Multi-organ chips to study endocrine interactions between the uterus and other organs

As we all know, successful implantation of an embryo and subsequent pregnancy require highly coordinated communication between the endometrium and ovaries. On the one hand, the endometrium, like the fallopian tube, is also regulated by ovarian hormones. Ovaries can facilitate embryo implantation by secreting estrogen and progesterone, altering the expression, local immune response, and secretory activity of some adhesion molecules in the endometrium [66]. On the other hand, the endometrium produces large amounts of prostaglandin E2, stimulating oocyte maturation and follicle rupture in the ovary [67]. There have been attempts to develop new in vitro culture models to reflect this bidirectional endocrine interaction between the endometrium and ovary. *Park et al.* created a dual reproductive organ chip that connects the ovarian and endometrial chambers to each other via medium channels, and allows for endocrine interaction between chambers through the diffusion of various hormones or cytokines. The endometrial chamber in the chip comprises human ESCs, stromal cells, and vascular endothelial cells, while the ovarian chamber incorporates human granular cells and follicular cells, simulating the multicellular complexity of the female reproductive system [68] (Fig. 5E). The platform was also combined with a luciferase reporting system for reproductive toxicity testing of hazardous substances.

Previously, *Edington et al.* fabricated a microfluidic platform with 10 organs, including the endometrium in addition to the heart, liver, kidney etc. The platform maintained the phenotypic function of all 10 modules for 4 weeks by controlling the circulating media through a microfluidic system that allowed them to interact and exchange endogenously produced molecules, and exemplifying signaling interactions between the endometrium and other organs [69].

Compared with single-organ chips, multi-organ chips can better reflect the physiological and pathological states of the human body and provide more accurate models for drug development, toxicity testing, and disease research. However, the research on multi-organ chips faces a number of technical difficulties that have led to its relatively slow development [70]. The first is the complexity of organ selection and combination. Different research objectives require different combinations of organs. For example, the study of drug absorption, distribution, metabolism and excretion requires organs such as intestines, livers and kidneys, while the study of cancer metastasis requires organs such as tumors, blood vessels and lymphatics. In addition, combining multiple organs requires consideration of their connection methods, fluid circulation, and signal transmission to ensure the functionality and stability of the multi-organ chip, such as the size, position, orientation, and interface of the organ model, and the parameters of the medium such as flow rate, pressure, temperature, and pH, which greatly increase the challenges of multi-organ chip integration and operation. Moreover, there are no unified methods and standards for constructing and evaluating these organ models, and different laboratories may use different cell sources, culture conditions, stimulation methods, etc., leading to differences in the quality and reproducibility of organ models.

The main advantage of these uterus and endometrium organ-on-a-chip systems is their ability to reproduce physiological conditions, such as cyclic estrogen and progesterone effects, shear stress due to dynamic flow in microfluidic systems, and bidirectional paracrine interactions between cells. They can be used as embryo culture platforms for assisted in vitro fertilization and are expected to be a powerful tool for assisted human reproduction. However, difficulties in obtaining primary cells and maintaining long-term in vitro culture remain major obstacles to research. In the future, more cell types, hormonal fluctuations under physiological conditions and more types of organs will be integrated into the microarrays to simulate the complex behavior of the human uterus and endometrium.

#### Cervical-vagina (Table 1, rows 16–19)

The cervix is the lower part of the uterus that connects to the vagina. They form a barrier between the uterine cavity and the external environment, protecting the uterus from infections. They also play important roles in pregnancy and childbirth. The cervix keeps the fetus inside the womb until it is ready to be born, and the vagina serves as the birth canal during labor [71]. The OOC technology for the cervix and vagina is relatively new and has gained more attention in recent years.

**Fig. 6:**
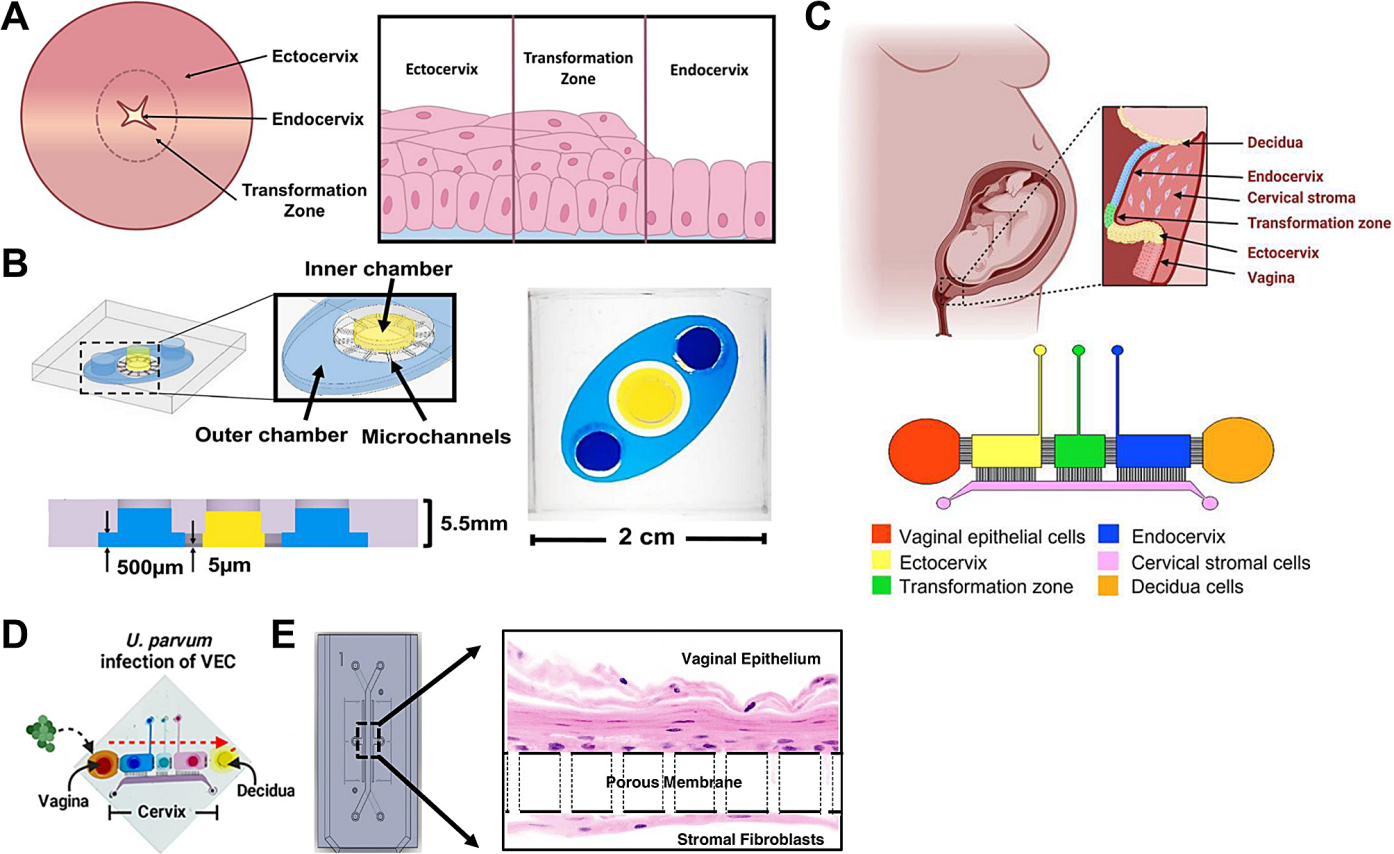
(**A**) Schematic representation of the anatomy of the cervical epithelial layer. Left: gross morphology view; Right: cross-sectional view. From outside to inside are ectocervix, transformation zone, endocervix. Reproduced with permission [73]. Copyright 2021 Federation of American Societies for Experimental Biology. (**B**) Design and cell culture of the cervix-organ-o-a-chip, the blue outer chamber cultures the ectocervical epithelial cells and the yellow inner chamber cultures the endocervical epithelial cells. The two cell culture chambers are separated by microfluidic channels filled with type IV collagen, through which the cells can migrate to mimic the epithelium of the transformation zone of the cervix. Reproduced with permission [73]. Copyright 2021 Federation of American Societies for Experimental Biology. (**C**) Upper: an illustration of the anatomy and histology of the female reproductive tract showing the vagina, cervix, and decidua. The epithelial cells of the vagina are continuous with the ectocervix, transformation zone, and endocervix. Beneath the epithelial layers are the cervical stromal layer embedded in collagen. During term gestation, the fetal membrane, specifically the decidua, which is its outermost layer, lies directly above the endocervix; Lower: schematic image of the vagina-cervix-decidua (VCD)-OOC with different cell culture chambers represented by different colors and connected with each other by an array of microchannels. Reproduced with permission [75]. Copyright 2022 Tantengco, Richardson, Radnaa, Kammala, Kim, Medina, Han and Menon. (**D**) Inoculation of *U.parvum* in the vaginal epithelial cells chamber of the VCD-OOC. The red arrow indicates the direction of propagation of *U. parvum* infection from the vaginal epithelial cells chamber to the decidual cell chamber. Reproduced with permission [74]. Copyright 2022 Federation of American Societies for Experimental Biology. (**E**) Schematic representation of the microfluidic human vagina-on-a-chip model. Human vaginal epithelial cells cultured in the top channel of the porous membrane and human uterine fibroblasts cultured in the submembrane channel, thus reconstructing the vaginal epithelial-stromal interface in vitro. Reproduced with permission [77]

The cervical canal is lined by the epithelial layer, which is divided into three distinct regions: ectocervix, transformation zone, and endocervix (Fig. 6A). This layer plays an important role in maintaining the overall health of the cervix, protecting the cervical matrix from pathogens present in the lower reproductive tract [72]. However, research on the mechanisms and pathophysiological effects of cervical bacterial infection during pregnancy has been limited due to the difficulty in obtaining cervical samples. *Tantengco’s* team has been working on etiological studies of the occurrence of premature birth caused by disruption of the cervical-vaginal barrier by infection or inflammation in the lower genital tract. They first created a cervix-on-a-chip (CE-OOC) model that enables co-culture of ectocervical and endocervical epithelial cells in two different but interconnected microenvironments [73]. This CE-OOC is characterized by an array of microfluidic channels filled with type IV collagen that connects the outer lumen (ectocervical epithelial cells) and the inner lumen (endocervical epithelial cells), which figuratively corresponds to the three different regions of the epithelial layer lining the cervical canal (Fig. 6B). They used the model to study the interaction of epithelial cells from different regions of the cervical epithelium under normal, infectious and/or inflammatory conditions, to further understand the important functions of the cervical epithelium and its influence on cervical remodeling during pregnancy. However, this CE-OOC lacks the cervical stromal cells that are needed to regulate the mechanical and tensile strength of the cervix. Therefore, to create a more physiologically relevant cervix-on-a-chip, they developed a vagina-cervix-decidua-organ-on-a-chip (VCD-OOC) to model the interface between the human vagina, cervix, and metaphase (Fig. 6C). It contains six micro-channelically interconnected cell culture chambers that culture cells from the vagina, ectocervix, transformation zone, cervical stroma, endocervix, and decidua cells, where the cervical stromal cell chambers and decidua cell chambers are connected to mimic the human fetal-maternal interface [74]. They also inoculated *U. parvum* in the vaginal epithelial cell (VEC) chambers of the VCD-OOC, and with the flow of medium, the pathogen gradually diffused into the decidua chambers, thus simulating an upstream infection from the lower vagina to the decidua [74] (Fig. 6D). Subsequently, their team used this model to further determine that exosomes from cervical ectodermal cells infected with *U. parvum* could carry bacterial antigens to cause inflammation at the fetal-maternal interface, but not enough to induce premature birth [75].

The vaginal environment, which is composed of host cells, symbiotic bacteria, pathogens, secretions, mucous membranes, immune factors, and other complex interactions, has attracted the attention of many scientists. The dysregulation of the female vaginal microbiota (VMB) affects the mucosal barrier, alters the mucus and epithelial cytoskeleton, and increases the levels of proinflammatory cytokines [76]. Most of our knowledge of VMB comes from genomic and metagenomic analyses of clinical samples. However, it has been difficult to study how VMB interacts with the human vaginal epithelium under physiologically relevant microenvironmental conditions. *Mahajan et al.* used OOC technology to engineer a microfluidic culture device lined with hormone-sensitive primary vaginal epithelial cells connected to stromal fibroblasts below, to reconstitute the vaginal epithelial-stromal interface in vitro (Fig. 6E). They maintained a low physiological oxygen concentration in the epithelium chamber of the vaginal mucosal OOC for assessing colonization of the optimal *L. crispatus* consortia as well as non-optimal Gardnerella vaginalis-containing consortia, and measure the related host innate immune response [77]. The present vaginal mucosal OOC may represent a human in vitro preclinical model that can be used to advance vaginal host microbiome research and accelerate the development of microbiome targeted therapies.

In addition, mucus, as a protective covering of many epithelial surfaces, can symbiotically balance with the vaginal microbiome and plays a vital role in the body’s defense mechanisms [78]. Mucus secreted by cervical epithelial cells flows into the vagina, but to date, the study of human cervical vagina mucus has been challenging due to the limitation of collection methods and the variability of subjects. OOC technology has been shown to model mucus physiology in vitro, and the mucus harvested from the chip can be used for follow-up tests including mucus biochemistry and mucus structure [78]. However, at present, the cervix-on-a-chip for cervical mucus are mostly used to study the movement patterns of male sperm, providing a platform for sperm selection in clinical infertility diagnosis [79]. The application of cervical vaginal mucus-related OOC model in female reproductive tract health and diseases has not been developed yet, which will be a new direction for the development of cervical vaginal OOC in the future.

### Organ-on-a-chip model of pregnancy

Pregnancy is a highly coordinated process, in which the female reproductive system undergoes a complex and interdependent series of physiological events. Modeling these different stages of pregnancy is an important goal of reproductive research and has important clinical significance. Many efforts have been made to deepen the understanding of the cellular and molecular events underlying trophoblast implantation and placental remodeling in early pregnancy, which play a pathogenic role in the development of pregnancy complications, such as preeclampsia and fetal growth retardation [80]. The ability to model and explore trophoblast invasion and implantation processes contributes greatly to the development of new drugs and treatment strategies for these serious diseases. The nutrient and gas transport and barrier function of the placenta in the middle and third trimester of pregnancy can protect the fetus from harmful pathogens and potentially toxic substances circulating in the maternal blood, and predict drug safety during pregnancy. Modeling the maternal-fetal interface and its functions has always been the focus of attention. Currently, common OOC in this field includes: (i) placenta-on-a-chip; (ii) fetal membrane-on-a-chip. This section highlights how placenta-on-a-chip is being used to address the technical challenges associated with in vitro modeling of these complex reproductive processes.

#### Placenta-on-a-chip

The placenta plays an indispensable role in supporting fetal development and is the key to successful reproduction [81]. Due to the lack of a suitable in vitro placental model systems, little is known about the pharmacokinetics of nutrients and drugs across the human placental barriers, leading to great uncertainty about the safety of many drugs during pregnancy. For ethical considerations, researchers are prohibited from using human placenta during pregnancy, so there is a long history of research to develop human placenta models in vitro. Current studies on placental structure and function mainly rely on animal [82] and transwell cell culture insert-based models [83], but none of these in vitro models can reliably mimic the human physiological characteristics of the maternal-fetal interface and replicate the multilayered structure of the placental barrier [84]. More complex and representative human placenta micro-engineered OOC models are presented here and can be roughly divided into three groups: (i) OOC focusing on placental transport function; (ii) OOC focusing on placental barrier function; (iii) OOC focusing on the invasive function of placental trophoblast cells.

##### **OOC focusing on placental transport function (**Table 2, **rows 1–7**)

**Table 2:** Organ-on-a-chip for studying pregnancy-related phenomena (focusing on placenta-on-a-chip)

Model category	Cell types	Culture environment	Device characteristics	Culture characteristics	Significance	Year/Reference
Placenta-on-a-Chip	λ Human trophoblasts (JEG-3) λ HUVECs	λ Medium: JEG-3: DMEM + 10% FBS + 1% penicillin/streptomycin; HUVECs: EBMTM-2 basal medium + Endothelial Cell Growth Medium-2 MV Bullet Kit λ Temperature: 37 °C; CO_2_: 5%	λ Two PDMS slabs containing microchannel features: 500 μm × 200 μm (width × height) λ A vitrified collagen membrane λ Device material: PDMS λ Fabrication method: soft lithography technology	λ Flow rate: 30 µl/h λ Coating: 40 µg/ml of fibronectin and 1.5% gelatin	Transport function of placental barrier—lucos transport	2016/ [16]
Placenta-on-a-Chip	λ Human trophoblasts (BeWo) λ HUVECs	λ Medium: Bewo: Ham’s F-12 K + 10% FBS + 40 µg/mL gentamicin; HUVECs: endothelial cell growth medium kit	λ Two PDMS microchannels features: 1 mm× 200 μm (width × height) λ A polycarbonate membrane containing 400 nm pores λ Device material: PDMS λ Fabrication method: soft lithography technology	λ Flow rate: 2 µl/h λ Coating: collagen I	Transport function of placental barrier—glucose transport	2020/ [86]
Placenta-on-a-Chip	λ Human trophoblasts (BeWo) λ HPVECs	λ Medium: BeWo: DMEM/F-12 K + 10% FBS + 1% L-glutamine + 1% penicillin/streptomycin; HPVECs: EGM-2 medium + 2% FBS λ Temperature: 37 °C; CO_2_: 5%	λ Two PDMS slabs containing microchannel features: 1.5 cm×1 mm×135 μm λ A vitrified collagen membrane λ Device material: PDMS λ Fabrication method: soft lithography technology	λ Flow rate: 100 µl/h λ Coating: 0.1 mg/ml human fibronectin solution	Transport function of placental barrier—glucose transport	2016/ [87]
Placenta-on-a-Chip	λ Ditto	λ Ditto	λ Ditto	λ Ditto	Transport function of placental barrier—drug transport (Glyburide)	2018/ [88]
Placenta-on-a-Chip	λ Ditto	λ Medium: BeWo: Ham’s F-12 K + 10% FBS; HUVECs: EBM + endothelial cell growth supplement + FBS λ Temperature: 37 °C; CO_2_: 5%	λ Two PDMS slabs containing microchannel features: 400 μm × 100 μm (width × height) λ A 0.4micron pore-sized polyester track etched membrane λ Device material: PDMS λ Fabrication method: soft lithography technology	λ Flow rate: 50 µl/h λ Coating: /	Transport function of placental barrier—caffeine transport	2019/ [89]
Fetal membrane decidua-on-a-Chip (FMi-OOC) and placenta-on-a-Chip (PLA-OOC)	λ Human decidua and fetal membrane cell λ Human placenta cell line	λ Medium: DEC: DMEM/F-12 + 10% FBS + 10% penicillin/streptomycin + 10% amphotericin B; AEC: KSFM medium + 30 µg/mL bovine pituitary extract + 0.1ng/mL epidermal growth factor + 0.4 mM CaCl_2_ + 0.5 mg/mL primocin; AMC + CMC: DMEM/F-12 + 5% FBS + 10% penicillin/streptomycin + 10% amphotericin B; CTC: DMEM/F-12 + 0.20% FBS + 0.1 mM β-mercaptoethanol + 0.5% penicillin/streptomycin + 0.3% BSA + 1× ITS-X + 2 µM CHIR99021 + 0.05 µM A83-01 + 1.5 µg/mL l-ascorbic acid + 50 ng/mL epithelial growth factor + 0.08 mM VPA + 1× Revitacell λ Temperature: 37 °C; CO_2_: 5%	λ FMi-OOC: four concentric-shaped culture compartments (one for maternal cells and three for fetal cells) λ PLA-OOC: STB + CTB + HUVEC chambers: 250 μm in height λ Device material: PDMS λ Fabrication method: soft lithography technology	λ Flow rate: / λ Coating: type IV basement membrane collagen Matrigel and collagen type 1	Transport function of placental barrier—drug transport and metabolism (Statin)	2022/ [90]
Placental barrier on-a-chip	λ Human trophoblasts (BeWo) λ HPVECs	λ Medium: BeWo: DMEM + 10% FBS + 1% penicillin-streptomycin; HUVECs: complete human endothelial cell medium λ Temperature: 37 °C; CO_2_: 5%	λ The top and bottom perfusion lanes features: 300 μm × 220 μm (width × height) λ The middle lane features: 350 μm × 220 μm (width × height) λ A meniscus pinning barrier λ Device material: PDMS λ Fabrication method: soft lithography technology	λ Flow rate: / λ Coating: /	Transporter function, barrier function and hormone secretion	2023/ [94]
Placenta-on-a-chip	λ Ditto	λ Medium: BeWo: DMEM/F-12 + 15% FBS + 1× L-Glutamine + 1% penicillin-streptomycin; HUVECs: ECM medium λ Temperature: 37 °C; O_2_: 95%; CO_2_: 5%	λ The top and bottom microchannels features: 2 mm×350 μm×200 μm λ The middle matrix channel features: 2 mm×300 μm×50 μm λ Device material: PDMS λ Fabrication method: soft lithography technology	λ Flow rate: 20 µl/h λ Coating: Collage I	Barrier function of placental—TiO_2_ NPs	2019/ [96]
Placental Nanoparticle Uptake-On-a-Chip	λ BeWo	λ Medium: BeWo: Ham’s F-12 K + 10% FBS + 1% L-Glutamine + 1% penicillin–streptomycin–neomycin λ Temperature: 37 °C; CO_2_: 5%	λ The microfluidic channel: 50 mm×5 mm×450 μm λ Device material: / λ Fabrication method: /	λ Flow rate: 22.89µL/min λ Coating: gelatin	Barrier function of placental—Placental Nanoparticle Uptake	2022/ [98]
Placenta-on-a-Chip	λ Human trophoblasts (BeWo) λ HVTs	λ Medium: BeWo: Ham’s F-12 + 10% FBS + 50 µg/ml kanamycin sulfate; HVTs: trophoblast medium + FBS + trophoblast growth factors λ Temperature: 37 °C; CO_2_: 5%	λ The upper layer channel: 15 mm×2 mm×200 μm λ The bottom layer channel: 20 mm×2 mm×200 μm λ The VC membrane: 10 μm thickness λ Device material: PDMS λ Fabrication method: soft lithography technology	λ Flow rate: 5 µl/h λ Coating: /	Intrusion function: to provide molecular insight into the microvilli-mediated mechanoresponsive cellular functions	2015/ [104]
Placenta-on-a-Chip	λ Human trophoblast cells λ dNK cells	λ Medium: tophoblast cells: DMEM + 20% FCS + 1 mM sodium pyruvate + 1× MEM non-essential amino acids + 2 mM L-glutamine, 10 units/ml penicillin + 100 µg/ml streptomycin + 2 mg/ml gentamycin; dNK cells: RPMI1640 + antibiotics + 10% FCS and 2.5 ng/ml IL-15 λ Temperature: 37 °C; CO_2_: 5%	λ The dimensions of each device: 4.5 × 2.3 cm with the length, width and height of each channel of 20 300 μm, 1300 μm and 150 μm respectively λ Device material: PDMS λ Fabrication method: soft lithography technology	λ Flow rate: 50 µl/h λ Coating: Matrigel	Intrusion function: to quantify the migratory characteristics of primary trophoblast cells to model EVT migration	2017/ [105]
Placenta-on-a-Chip	λ Human first trimester placenta cells (HTR8/SVneo) λ HUVECs	λ Medium: HTR8/SVneo: DMEM/F-12 + 1% penicillin–streptomycin + 2 mM L-glutamine + 10% FBS + 10 mM HEPES; HUVECs: complete medium + EGM-2 Plus medium λ Temperature: 37 °C; CO_2_: 5%	λ The center of the 3D microfluidic chip with the central compartment, outer channels (width: 200 μm), and pillar barrier (width: 50 μm), filled with pillars (pillar spacing: 3 μm) λ Device material: PDMS λ Fabrication method: /	λ Flow rate: 0.01/0.05/0.1 µl/min λ Coating: extracellular matrix	Intrusion function: to recreate the placental invasion microenvironment	2021/ [106]
Placenta-on-a-Chip	λ hiPSC	λ Medium: mTeSR1 medium λ Temperature: 37 °C; CO_2_: 5%	λ The first layer: PMMA, 90 × 50 × 4 mm^3^ λ The second layer: PDMS, 76 × 26 × 4 mm^3^ λ The third layer: PDMS, 76 × 26 × 1.5 mm^3^ λ The fourth layer: PMMA, 90 × 50 × 5 mm^3^ λ Device material: PMMA and PDMS λ Fabrication method: laser cutting and customized cutting die technology	λ Flow rate: 1 ml/min λ Coating: 0.5 mm thick Matrigel	Intrusion function: to create an in vitro placental trophoblast-like model with major cell types of the human placenta, including CTBs, differentiated subtypes, STBs, and EVTs	2022/ [107]
Placenta-on-a-Chip	λ hTSCs	λ Medium: hTSCs: TS medium + DMEM/F-12 + 0.1 mM 2-mercaptoethanol + 0.2% FBS + 0.5% Penicillin–Streptomycin + 0.3% HAS + 1% ITS-X supplement + 1.5 mg/mL L-ascorbic acid + 50 ng/mL EGF + 2 mM CHIR99021 + 0.5 mM A83-01 + 1 mM SB431542 + 0.8 mM VPA + 5 mM Y27632; HUVECs: Collagen I-coated plates and maintained in endothelial cell medium (ECM) λ Temperature: 37 °C; CO_2_: 5%	λ The upper and lower layers with microchannel: 20 × 1.2 × 0.2mm^3^ λ Device material: PDMS λ Fabrication method: soft lithography technology	λ Flow rate: 10µL/h λ Coating: hTSCs: Collagen IV; HUVECs: Collagen I	Intrusion function: to describe a biomimetic placental barrier model of hTSCs in a perfused organ chip system	2023/ [108]

**Notes**: HVTs, human villous trophoblasts; HUVECs, human umbilical vein endothelial cells; DMEM, Dulbecco’s Modified Eagle Medium; FBS, fetal bovine serum; HPVECs: Human primary placental villous endothelial cells; EBM, endothelial basal medium; DEC: decidua parietalis cells; AEC: amnion epithelial cells; AMC: amnion mesenchymal cells; CMC: chorion mesenchymal cells; CTC: chorion trophoblast cells; ECM, endothelial cell medium; FCS: fetal calf serum; IL-15, Interleukin 15; EVT, extra villous trophoblast; PMMA, polymethyl methacrylate; hiPSC, Human Pluripotent Stem Cell; CTBs, trophoblast progenitor cytotrophoblasts; STBs, syncytiotrophoblasts; hTSCs, human trophoblast stem cells

**Fig. 7:**
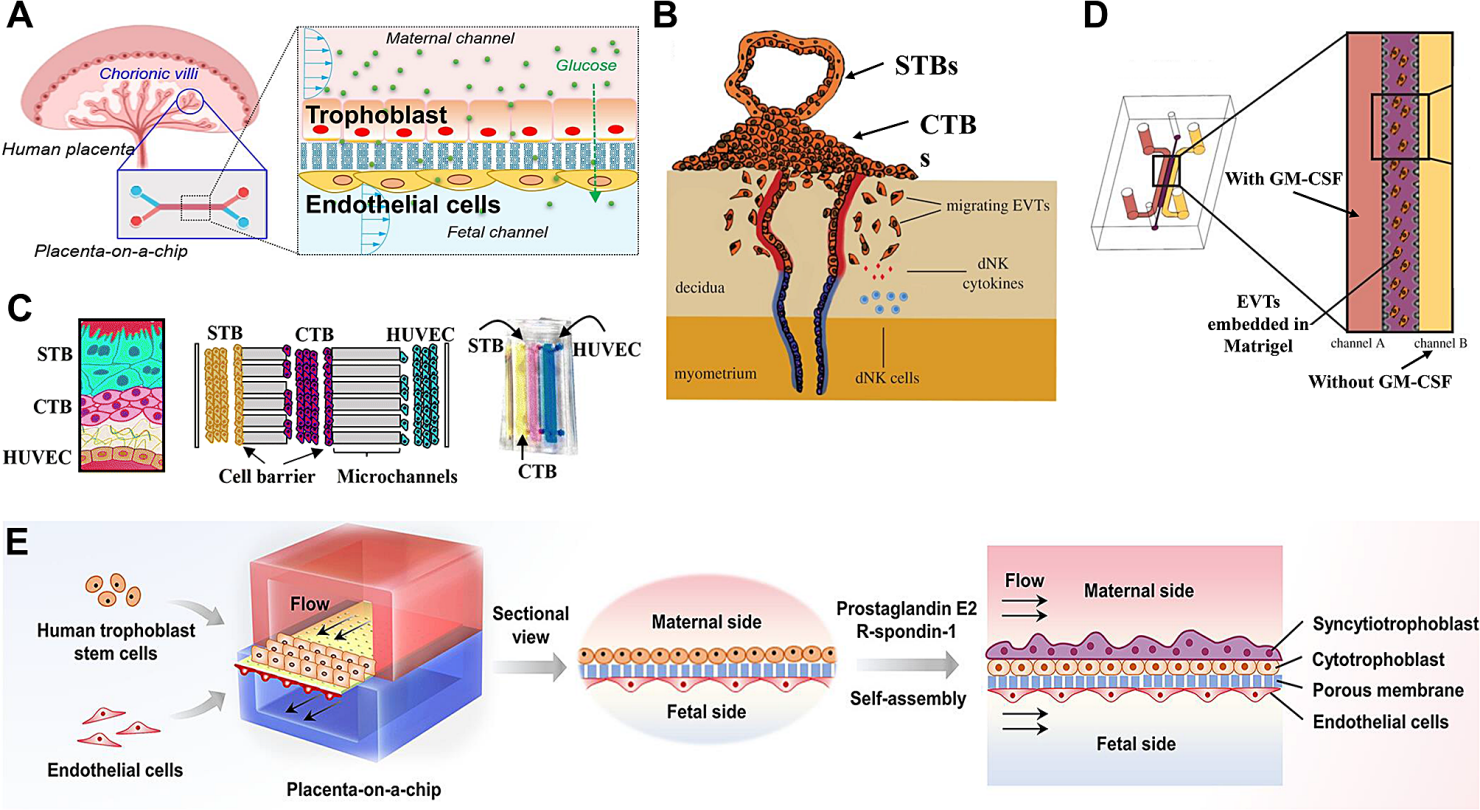
Mimicking placental function on the chip. (**A**) Schematic of the human placenta chip model. Human umbilical vein endothelial cells (HUVECs) and trophoblast cells were co-cultured to mimic the placental barrier interface between the mother and the fetus. Reproduced with permission [86]. Copyright 2020 Mosavati, Oleinikov and Du. (**B**) The placenta is implanted in the maternal decidua during the first trimester of pregnancy, with syncytiotrophoblasts (STBs) located on the outer surface of the placenta in direct contact with the maternal blood, and cytotrophoblasts (CTBs) located on the inner surface of the placenta. Fetal extravillous trophoblasts (EVTs) detach from the placenta and invade the maternal decidua to remodel the uterine spiral arteries. Maternal leukocytes present at the maternal-fetal interface, including decidual natural killer (dNK) cells, may regulate trophoblast invasion and spiral artery transformation through the secretion of cytokines (e.g., GM-CSF). Reproduced with permission [105]. Copyright 2017 Abbas, Oefner, Polacheck, Gardner, Farrell, Sharkey, Kamm, Moffett and Oyen. (**C**) Schematic of the placenta trophoblast-endothelial interface and placenta organ-on-a-chip (PLA-OOC). The PLA-OOC contains three rectangular cell culture chambers separated by arrays of microchannels. The cells are seeded as follows, from left to right: syncytiotrophoblasts (yellow), the center chamber contains cytotrophoblasts (pink), and the right chamber contains human umbilical vein endothelial cells (HUVECs) forming the endothelial layer (blue). Reproduced with permission [90]. Copyright 2022 Richardson, Kammala, Costantine, Fortunato, Radnaa, Kim, Taylor, Han and Menon. (**D**) Microfluidics as a model for trophoblast invasion. EVTs are isolated from first trimester placentas, embedded in growth factor-reduced Matrigel in the central hydrogel channel. A constant flow of medium is applied in the two side channels, one with (channel A) and without GM-CSF (channel B) to create a gradient of the cytokine across the hydrogel channel. Reproduced with permission [105]. Copyright 2017 Abbas, Oefner, Polacheck, Gardner, Farrell, Sharkey, Kamm, Moffett and Oyen. (**E**) A bioengineered placental barrier model was constructed in a perfused organ-on-a-chip system. The human trophectodermal stem cells were inoculated on the upper channel, where they could differentiate into cytotrophoblasts and syncytiotrophoblasts and self-assemble into a double-layered trophoblast epithelium with a placental microvillus-like structure under dynamic culture conditions. Human umbilical vein endothelial cells were cultured on the other side of the collagen-coated membrane to mimic the fetal endothelium. Reproduced with permission [108]. Copyright 2023 Cao, Wang, Liu, Rong and Qin

In 1999, *Ma et al.* developed the first in vitro placenta model using a fiber-based perfusion bioreactor system to cultivate human trophoblasts. The results showed that tissue-engineered human trophoblast cells were feasible for the development of a drug detection model system in a perfusion bioreactor system [85]. Later, *Lee et al.* co-cultured the human trophoblast JEG-3 cell line and HUVECs, and first proposed the concept of placenta-on-a-chip (PLA-OOC) to test the physiological function of micro-engineered placental barriers by measuring glucose transport across the trophoblast-endothelial interface [16]. Similar PLA-OOC models have been developed to investigate the feasibility of crossing the human placental barriers (Fig. 7A), including glucose [86, 87], glyburide [88], caffeine [89], and statins [90], providing impetus to improve and innovate traditional models of reproductive toxicology. These PLA-OOC models co-culture endothelial and trophoblast cells in close proximity on either side of an intermediate porous membrane and provide dynamic flow conditions to mimic the placental barrier composed of trophoblast-endothelial cells. Trophoblast cells are the main component of the placenta, where mononuclear cytotrophoblast cells (CTBs) can differentiate into multinucleated syncytiotrophoblasts (STBs) and extravillous trophoblasts (EVTs) (Fig. 7B) [91]. The PLA-OOC developed by *Lauren et al.* appeared to be more fine-grained because it included CTBs, STBs, and HUVECs (Fig. 7C) [90]. The fetal endothelial cell chamber in this PLA-OOC was connected to the CTBs chamber by an array of 24 microchannels that were coated with type I collagen to mimic the matrix of the placenta.

In addition, the placental barrier facilitates gas exchange between the mother and fetus [92], so the function of the artificial placenta in gas transport has been investigated. For example, a high-performance, pumpless artificial placenta microfluidic oxygenator with a double-sided single oxygenator unit was used to support the oxygen requirements of preterm newborns [93]. Recently, *Rabussier et al.* constructed an in vitro model of the placental barrier using trophoblast and endothelial cells, and exposed the model to hypoxic conditions and modulated perfusion flow to induce a hypoxic pathological environment in the uteroplacental [94]. They believe that this model can help to mechanistically understand preeclampsia and other hypoxia/ischemia-related placental pathologies and support the development of effective future therapies through target and compound screening activities.

In summary, the current research on the structures of selective transport in the placenta can serve two purposes: to validate the successful construction of PLA-OOC on one hand, and to screen and develop drugs for safety verification on the other hand.

##### **OOC focusing on placental barrier function** (Table 2, **rows 8–9**)

Microfluidic organ chips have also shown potential in the application of maternal-fetal barrier function, providing a new opportunity to screen and understand the protective role of the placental barriers for the fetus. The commercialization of nanomaterials has raised great concerns about the long-term exposure of pregnant women to nanoparticles (NPs) [95]. *Yin et al.* constructed a model that incorporated trophoblasts, basement membrane stroma, endothelial cells, and monocyte-derived macrophages into a microfluidic system. They introduced TiO2-NPs on the maternal side of the chip to model the toxic effects of environmental exposure of NPs on the human placental barrier [96]. *Schuller et al.* integrated biosensors onto porous PET membranes to create a novel lab-on-a-chip platform to determine the toxicity of SiO_2_, TiO_2_, and zinc oxide NPs to placental cytotrophoblasts (BeWo cell) [97]. Although this platform is not a microfluidic OOC platform, the integration of biosensors and microfluidic technology may lead to a new phase of research in this field in the near future. *Abostait’s* group designed a dynamic placental chip using BeWo cells to study the effect of trophoblast layer fusion and microvillus formation during pregnancy on the cellular uptake of liposomes, demonstrating significant differences in the extent of liposome uptake under different conditions [98]. Placental chip models for fetal risk assessment have been reviewed extensively [99–101], and will not be discussed in detail in this section.

Together, these laboratory models of human placenta-on-a-chip provide a platform to study the maternal-fetal effects of NPs exposure and have great advantages in terms of characterization, transport, and degradation. In the future, as technology progresses, there will be significant improvements in bionics, mechanical strength, and cell junction complexity, which will enable a better assessment of drug safety during pregnancy.

##### **OOC focusing on the invasive function of placental trophoblast cells** (Table 2, **rows 10–14**)

About 5-6 days after fertilization, the developing embryo enters the decidualized uterus from the fallopian tube, where it adheres to and invades the endometrial epithelium and stroma, a key reproductive event known as embryo implantation [102]. Later, the outermost layer of the embryo gives rise to the cytotrophoblast, which forms placental villi that anchor the embryo to the maternal decidua through branching morphogenesis. During placentation, the trophoblasts at the anchoring villi differentiate into invasive cells called extra-villus trophoblasts (EVTs), and the adhesion and invasion of EVTs to the maternal uterine epithelium is essential for placentation [103] (Fig. 7B). These cells penetrate deep into the endometrial matrix, reach and remodel maternal blood vessels to establish the uteroplacental circulation that provides vascular supply for the growing fetus. Therefore, to understand normal and abnormal pregnancy, many researchers have focused on the physiological process of trophoblast invasion.

In 2015, *Miura et al.* constructed a novel placenta-on-a-chip, co-culturing human trophoblast Bewo cell line and human villous trophoblasts (HVTs), which demonstrated that the formation of microvilli is a prerequisite for the placental barrier to function as a transporter, and fluid shear stress is a key external trigger for microvillus formation [104]. *Abbas et al.* isolated EVTs from early gestational placenta and embedded them in growth factor-reduced Matrigel in a central hydrogel channel. A constant medium flow was applied in both channels, one with (channel A) and one without granulocyte-macrophage colony-stimulating factor (GM-CSF) (channel B), to create a cytokine gradient across the hydrogel channel. They used the device to examine how a soluble factor (GM-CSF) produced by resident immune cells (decidual natural killer cells) in the maternal decidua affects the directional migration of EVTs during placental development [105] (Fig. 7D). *Pu et al.* explored cell invasion using a PLA-OOC, which incorporated the HTR8/SVneo trophoblast cell line [106], and subsequently with advances in technology, human induced pluripotent stem cells (hiPSCs) and human trophectodermal stem cells (hTSCs) have begun to replace the trophoblast cell line in co-cultures with HUVECs cells for the purpose of probing the differentiation and invasion that occurs in these stem cells during embryo implantation. For example, *Deng et al.* created an in vitro placental trophoblast-like model using self-organization of hiPSCs, a device that allows in situ trophoblast lineage differentiation and enables hiPSCs to form 3D clusters with the major cell types of the human placenta, including CTBs, STBs, and EVTs in a bionic microenvironment [107]. This is the first study to generate hiPSCs-derived 3D placenta-like tissue models on perfusion chips by combining engineering strategies with developmental principles. This study also suggested an important role of fluid flow in promoting the trophoblastic differentiation, which is consistent with the findings of *Miura’s* [104] and *Abostait’s* [98] teams. In addition, hTSCs have been applied to the construction of bionic placenta models. *Cao et al.* co-cultured hTSCs obtained from primary placental tissues or blastocysts and HUVECs, and found that hTSCs could differentiate into CTBs and STBs, which self-assembled into a double-layer trophoblast epithelium with placental microvillus-like structures under dynamic culture, and formed a placental barrier that showed the presence of dense microvilli and better transport, endocrine and barrier functions [108] (Fig. 7E). In a word, this type of placental models focusing on simulating the function provides a better overview of the key structural and functional characteristics of early human placenta development in a physiologically relevant microenvironment.

In fact, most of the 3D PLA-OOC microsystems currently available can mimic the physiological microstructure (trophoblast invasion), transport and barrier function of the human placental barrier in vivo, but with slightly different focuses and purposes in the research process, and the three aspects cannot be completely separated. Overall, this type of PLA-OOC is powerful for in vitro studies of barrier integrity and the ability of molecules to across the maternal-fetal interface. Future directions for the improvement of such systems include: (1) To obtain a more complex model of the human maternal-fetal interface barrier by culturing trophoblast cells to differentiate into STBs and CTBs and incorporating more types of immune cell populations at the maternal-fetal interface (e.g., macrophages, T cells, NK cells, etc.) into the microfluidic system; (2) To investigate the transport of other nutrients, not only glucose, to further validate the physiological relevance of the model; (3) To assess the possibility of transmission of harmful substances such as bacteria and viruses through the barriers; (4) To optimize the flow rate of the placental microfluidic chip; (5) To simulate placental dysfunction known to be associated with pathological conditions, such as diseases like fetal growth restriction, preeclampsia, and recurrent miscarriage, for screening new drugs and developing alternative treatments.

#### Placental membrane-on-a-chip

The fetal membrane plays a key structural role in maintaining the fetal and maternal compartments of the pregnant uterus. When people refer to the fetal membrane, they mostly mean the chorionic membrane and the amniotic membrane [109]. Currently, the research on fetal membrane-on-a-chip mainly focuses on the effect of fetal membrane inflammation on preterm delivery and premature rupture of fetal membranes. Therefore, fetal membrane-on-a-chip will be described in detail in the section of organ chip modeling of inflammatory diseases.

## Application of OOC in pathophysiological modeling of female reproductive system diseases

OOC has emerged as a promising field to simulate complex human diseases and overcome the limitations of traditional animal and in vitro models for studying human reproductive diseases. In this section, we mainly focus on the pathophysiological models of the following three conditions: (i) inflammatory/infectious diseases; (ii) neoplasms of the reproductive system; (iii) other diseases, such as endometriosis and preeclampsia.

### **OOC model of inflammatory/infectious diseases (**Table 3, **rows 1–9)**

**Table 3:** Organ-on-a-chip for modeling the pathophysiology of female reproductive system diseases

Model category	Cell types	Culture environment	Device characteristics	Culture characteristics	Significance	Year/Reference
Placenta-on-a-Chip	λ HUVECs λ BeWo	λ Medium: HUVECs: ECM medium; BeWo: DMEM/F-12 medium; λ Temperature: 37 °C; CO_2_: 5%	λ The upper and lower layers with microchannels: 1.5 cm×1.5 mm×400 μm λ Transparent semipermeable membrane: 0.4 μm pores λ Device material: PDMS λ Fabrication method: soft lithography technology	λ Flow rate: 10 µl/h λ Coating: 0.1 mg/mL Collagen I	Placental inflammation model	2018/ [114]
Placenta-on-a-Chip	λ HUVECs λ BeWo	λ Medium: HUVECs: endothelial cell growth medium; BeWo: Ham’s F-12 K nutrient mixture + 10% FBS + 40 mg/mL gentamicin λ Temperature: 37 °C; CO_2_: 5%	λ Three channels: 300 μm wide and 220 μm high λ Device material: PDMS λ Fabrication method: soft lithography technology	λ Flow rate: / λ Coating: ECM gel	Placental inflammation model	2022/ [115]
Amnion membrane organ-on-chip	λ hiPSC	λ Medium: mTesR1 medium λ Temperature: 37 °C; CO_2_: 5%	λ Two parallel medium channels: 1 × 0.5 × 1.4mm^3^ λ The middle matrix channel: 1 × 0.2 × 1.4mm^3^ λ Device material: PDMS λ Fabrication method: soft lithography technology	λ Flow rate: 1 µl/h λ Coating: Matrigel	Amniotic inflammation model	2020/ [122]
Amnion membrane organ-on-chip	λ Primary AECs λ Primary AMCs	λ Medium: AECs: Ham’s F-12/DMEM + 10% FBS + 10 ng/ml EGF + 2 mM L-glutamine + 100 U/ml penicillin G + 100 mg/ml streptomycin; AMCs: complete DMEM-F12 medium + 5% FBS + 100 U/ml penicillin G + 100 mg/ml streptomycin λ Temperature: 37 °C; CO_2_: 5%	λ The first layer: 5 μm deep λ The second layer: 500 μm thick λ Device material: PDMS λ Fabrication method: soft lithography technology	λ Flow rate: / λ Coating: type IV collagen matrigel	Oxidative stress model	2019/ [116]
Amnion membrane organ-on-chip	λ AECs λ Decidual cells	λ Medium: AECs: Ditto; Decidual cells: Ham’s F-12/DMEM + 5% FBS + 10 ng/mL EGF + 100 U/ml penicillin G + 100 mg/ml streptomycin λ Temperature: 37 °C; CO_2_: 5%	λ Two microfluidic chambers: 4.75 mm×6.2 mm λ A semipermeable polycarbonate membrane λ Device material: PDMS λ Fabrication method: soft lithography technology	λ Flow rate: / λ Coating: Matrigel	Oxidative stress model	2020/ [117]
Feto-maternal interface organ-on-chip	λ AECs λ Decidua cells λ CTs λ AMCs	λ Medium: AECs: Ditto; Decidual cells: Ditto; CTs: Ham’s F12/DMEM + 10% FBS + 100 U/mL penicillin G + 100 mg/mL streptomycin; AMCs: DMEM-F12 medium + 5% FBS + 100U/mL penicillin G + 100 mg/mL streptomycin λ Temperature: 37 °C; CO_2_: 5%	λ Chamber 1: decidual cells, 250 μm in height, 3000 μm in width λ Chamber 2: fetal CTs and CMCs, 250 μm in height, 2000 μm in width λ Chamber 3: AMCs, 250 μm in height, 2000 μm in width λ Chamber 4: AECs, 250 μm in height, 600 μm in width λ Device material: PDMS λ Fabrication method: soft lithography technology	λ Flow rate: / λ Coating: type IV collagen	Maternal infection model	2020/ [118]
Feto-maternal interface organ-on-chip	λ Ditto	λ Ditto	λ Ditto	λ Ditto	Environmental toxin exposure model at the feto-maternal interface	2022/ [119]
Feto-maternal interface organ-on-chip	λ hFM_AEC λ hFM_DEC λ hFM_CTC λ hFM_AMC	λ Medium: hFM_AEC: KSFM + 30 µg/mL bovine pituitary extract + 0.1 ng/mL EGF + 0.4 mM CaCl2 + 0.5 mg/mL primocin; hFM_DEC: DMEM/F-12 + 10%FBS + 10% penicillin/streptomycin + 10% amphotericin B; hFM_CTC: DMEM/F-12 + 0.20% FBS + 0.01 mM β-mercaptoethanol + 0.5% penicillin/streptomycin + 0.3% BSA + 1× ITS-X + 2 µM CHIR99021 + 0.05 µM A83-01 + 1.5 µg/mL L-ascorbic acid + 50 ng/mL EGF + 0.08 mM VPA + 1×Revitacell; hFM_AMC: DMEM/F-12 + 5% FBS + 10% penicillin/streptomycin + 10% amphotericin B λ Temperature: 37 °C; CO_2_: 5%	λ Ditto	λ Ditto	Oxidative stress model at the feto-maternal interface	2023/ [120]
Amniotic fluid organ-on-a-chip	λ Amniotic fluid λ Human fetal brain cell	λ Medium: Human fetal brain cell: ATCC-formulated Eagle’s Minimum Essential Medium + 10% FBS λ Temperature: 37 °C; CO_2_: 5%	λ The first microchannel layer: 600 × 30 × 5µm^3^ λ The second cell culture chamber layer: 500 μm deep λ Device material: PDMS λ Fabrication method: soft lithography technology	λ Flow rate: / λ Coating: type IV collagen	Fetal neuroinflammation model	2022/ [121]
OvCa-Chip	λ A2780 λ HUVECs	λ /	λ The top tumor channel: 400 μm wide, 100 μm high λ The bottom vascular channel: 400 μm wide, 100 μm high; 20 mm long λ Device material: PDMS λ Fabrication method: soft lithography technology	λ Flow rate: 0.5 µL/min λ Coating: collagen-fibronectin	Ovarian cancer-vascular-blood model	2020/ [19]
OvCa-Chip	λ HOMECs λ A2780	λ Medium: HOMECs: RPMI 1640 + 100 U/ml penicillin + 100 µg/ml streptomycin; A2780: RPMI 1640 + 100 U/ml penicillin + 100 µg/ml streptomycin λ Temperature: 37 °C; CO_2_: 5%	λ The top portion of the device was changed from a single microchannel (1 mm×1 mm×100 μm) to three parallel microchannels λ Each micropillar: 250 × 100 × 100µm^3^ λ Device material: PDMS λ Fabrication method: soft lithography technology	λ Flow rate: / λ Coating: collagen I	Ovarian tumor microenvironment chip	2021/ [128]
Endometriosis organ-on-a-chip	λ ESCs λ HPMCs	λ Medium: ESCs: DMEM + 10% FBS + 2000 u penicillin + streptomycin + glutamine; HPMCs: complete medium λ Temperature: 37 °C; CO_2_: 5%	λ The microchannels: 1.5 cm×300 μm×300 μm λ Device material: PDMS λ Fabrication method: /	λ Flow rate: / λ Coating: /	Pathological model of peritoneal endometriosis	2012/ [14]
Endometriosis organ-on-a-chip	λ 12Z cells	λ Medium: DMEM-F12 medium λ Temperature: 37 °C; CO_2_: 5%	λ Device features: / λ Device material: PDMS λ Fabrication method: soft lithography technology	λ Flow rate: / λ Coating: collagen hydrogel	Organotypic organ-on-a-chip luminal model for endometriosis	2023/ [137]

Notes: AMCs, amnion mesenchymal cells; AECs, amnion epithelial cells; FBS, fetal bovine serum; EGF, epidermal growth factor; CTs, chorion trophoblasts; hFM_AEC, human amnion epithelial cells; hFM_DEC, human maternal decidual cells; hFM_CTC, human chorion trophoblast cells; hFM_AMC, human amnion mesenchymal cells; ESCs, Endometrial stromal cells; HPMCs, Human peritoneum mesothelial cells.

**Fig. 8:**
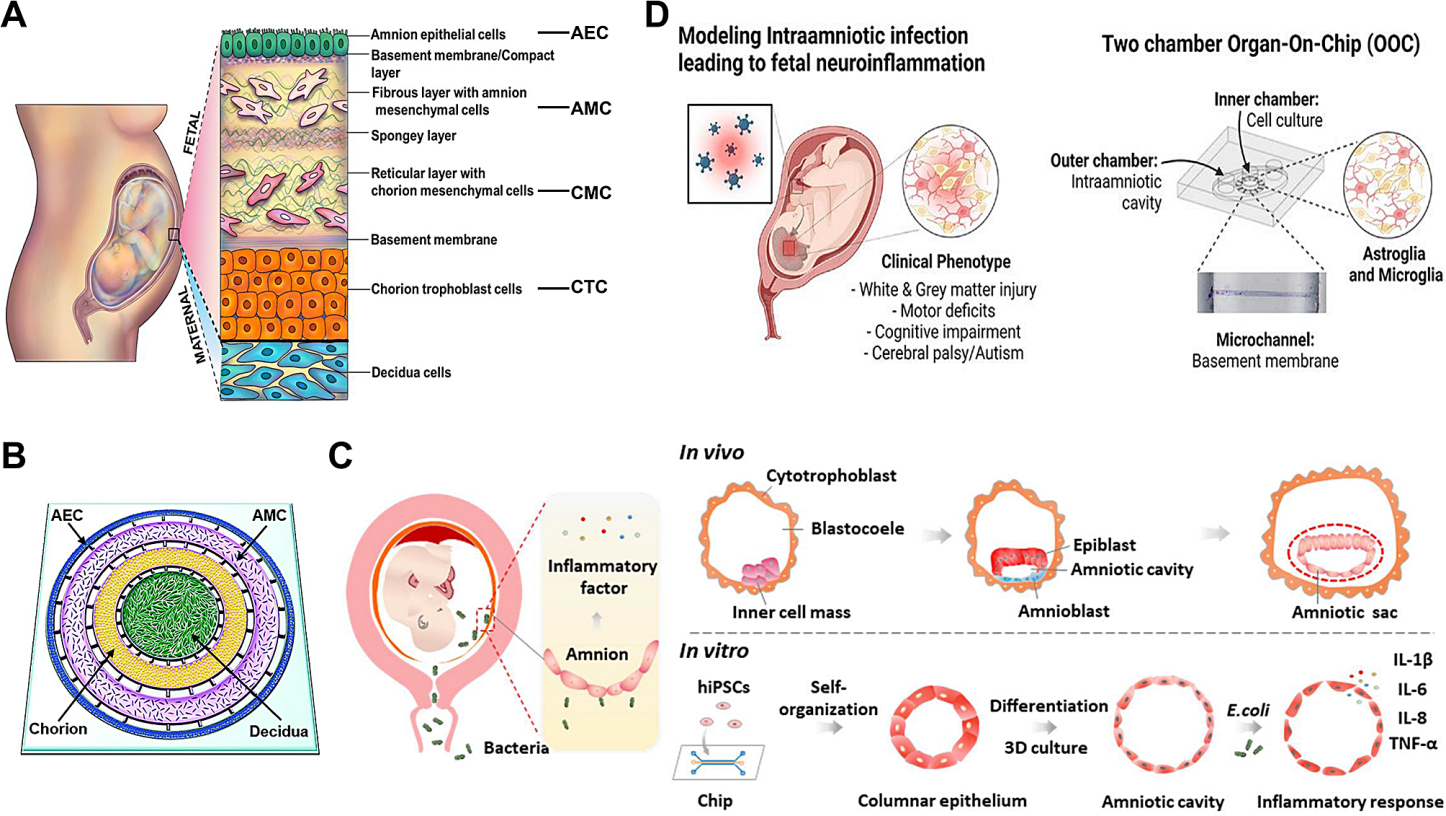
Mimicking inflammatory/infectious diseases on the chip. (**A**) Diagrammatic representation of the anatomy of the fetal membrane feto-maternal interface (FMi) with its cellular components, including amnion epithelial cells (AEC, green), amnion mesenchymal cells (AMC, light pink), chorion mesenchymal cells (CMC, dark pink), chorion trophoblast cells (CTC, orange), and maternal decidua cells (blue). Reproduced with permission [120]. Copyright 2023 Federation of American Societies for Experimental Biology. (**B**) Illustration of the feto-maternal interface organ-on-chip (FMi-OOC). The FMi-OOC contains four co-centric circular cell culture chambers connected by arrays of microchannels. The cells are seeded following the in vivo structure: amnion epithelium cells (AEC, blue), amnion mesenchymal cells (AMC, purple), chorion mesenchymal cells/chorion trophoblast cells (CMC/CTC, yellow), and decidua cells (green), respectively. Reproduced with permission [118]. Copyright 2020 Richardson, Kim, Han and Menon. (**C**) Schematic depiction of the amnion-on-a-chip device for the investigation of amniotic inflammatory responses under bacterial exposure. Intra-amniotic infection during pregnancy is associated with the inflammatory responses of amnion tissues to bacterial exposure. In vivo amniogenesis involves epiblast expansion, amnioblast differentiation, and amniotic formation during implantation of a human embryo. The developmental process of the amniotic tissue derived from human induced pluripotent stem cells (hiPSCs) by self-organization on a chip and its use to study amniotic inflammatory responses to *Escherichia coli (E. coli*) exposure. Reproduced with permission [122]. Copyright 2020 Yin, Zhu, Wang, Wang, Li and Qin. (**D**) Left: schematic description of the fetal brain phenotype resulting from an intraamniotic inflammation that can lead to fetal neuroinflammation. Right: schematic of the two-chamber OOC device showing the outer chamber, representing the intraamniotic cavity containing amniotic fluid with or without infectious stimuli, and the inner chamber, representing the fetal brain containing glial cells. Reproduced with permission [121]. Copyright 2022 John Wiley & Sons A/S. Published by John Wiley & Sons Ltd

The placenta consists of fetal tissues (amnion and chorionic villi) and maternal tissues (decidua) and is part of the fetal organs, which is an important site for material exchange, metabolism, hormone secretion and foreign microorganism invasion, and ensures normal development of the fetus [81]. The amniotic membrane covers the surface of the fetal side of the placenta and is composed of amnion epithelial cells (AEC) and amnion mesenchymal cells (AMC). The chorionic villus, also known as the villous chorion, is the main structure of the placenta and consists of two distinct cell types: chorion trophoblast cells (CTC) and chorion mesenchymal cells (CMC) (Fig. 8A). Preterm birth (PTB) is clinically defined as the delivery before 37 weeks of gestation [110]. Although the pathophysiology of PTB remains largely unknown, clinical studies have shown that cases of PTB often involve subclinical infections, such as chorioamnionitis, which typically manifests as inflammation of the chorion, amniotic membrane, and placenta [109]. This pathological condition releases a large number of proinflammatory mediators, which pose a great threat to fetal development and cause a systemic inflammatory response in the fetus [111]. Pathogenic microorganisms can even affect epigenetic mechanisms and lead to differential DNA methylation in the human placenta, thereby affecting fetal neurodevelopment [112]. However, due to the complex structure and function of the placenta and fetal membranes, the in-depth study of the underlying mechanisms is limited by traditional cell and animal models [113]. Here we discuss the OOC models that mimic chorioamnionitis during pregnancy.

*Zhu et al.* in 2018 fabricated a simple three-layered placental inflammation chip (i.e., trophoblasts/porous membrane/endothelial cells) and co-cultured it with Gram-negative *Escherichia coli* and examined the inflammatory response of endothelial cells infected with bacteria on the maternal side [114]. *Mosavati et al.* used a similar three-layer OOC to simulate the pathology of placental malaria (PM), using a theoretical model of biological mass transfer to analyze the impaired exchange of nutrients between the fetus and the mother in PM [115].

Besides placental inflammation, the inflammation of the feto-maternal interface (FMi) is also of great interest to scientists. *Richardson’s* team has been committed to this field for a long time, having first developed an amnion-on-a-chip that allows co-culture of AMC and AEC to monitor the migration and transition of amniotic cells [116]. They further co-culture AEC and decidual cells in OOC [117], revealing that dysregulation of oxidative stress (OS)-mediated cellular remodeling was associated with PTB. They went on to improve the OOC device, consisting of five concentric circles, each forming a cell culture chamber, each culturing one type of progenitor cell (decidua cells, chorionic villus cells (CTC/CMC), and amnion cells (AEC/AMC)), aiming to mimic the thickness and cell density of FMi in vivo [118] (Fig. 8B). They also collaborated with *Tantengco’s* team to study the ascending infection of *Ureaplasma parvum* in the female reproductive tract [74], and used the same model to study the varying degrees of adverse effects caused by the environmental toxins cadmium [119] and OS [120] on the fetal and maternal sides of the FMi. In addition, they alternatively took amniotic fluid from different patients and co-cultured it with neuroglia to establish an OOC model, demonstrating that fetal neuroinflammation is associated with intra-amniotic infection and inflammation associated with PTB [121] (Fig. 8C). *Yin et al.* established a micro-engineered human induced pluripotent stem cells (hiPSCs)-derived amniotic membrane chip. Under microfluidic culture conditions, hiPSCs self-renewed and differentiated into amniotic tissue and formed a well-defined amniotic cavity (Fig. 8D). By introducing *Escherichia coli* into the amniotic membrane chip to mimic intra-amniotic infection, they found that amniotic tissue exhibited significant dysfunction during amniotic membrane inflammation, including induction of apoptosis, disruption of cellular junctional integrity and increased secretion of inflammatory factors [122].

In these studies, we can see increasingly refined cell types being incorporated into OOC devices, with an increasing ability to mimic the pathology of inflammatory/infectious diseases. In the future, inflammatory cells (including granulocytes and T cells) as well as inflammatory factors secreted by the placenta can be attempted to be incorporated into the chip chambers. The complex crosstalk between pathogenic microorganisms and immune cells at the FMi of the organism will help to construct better models to predict the responses of local organisms at the FMi in pathological states. In addition, the physicochemical factors, mechanical strength and physiological thickness of the placental models are also major challenges for creating more complex and realistic placental models.

### **OOC model of cancers (**Table 3, **rows 10–11)**

**Fig. 9:**
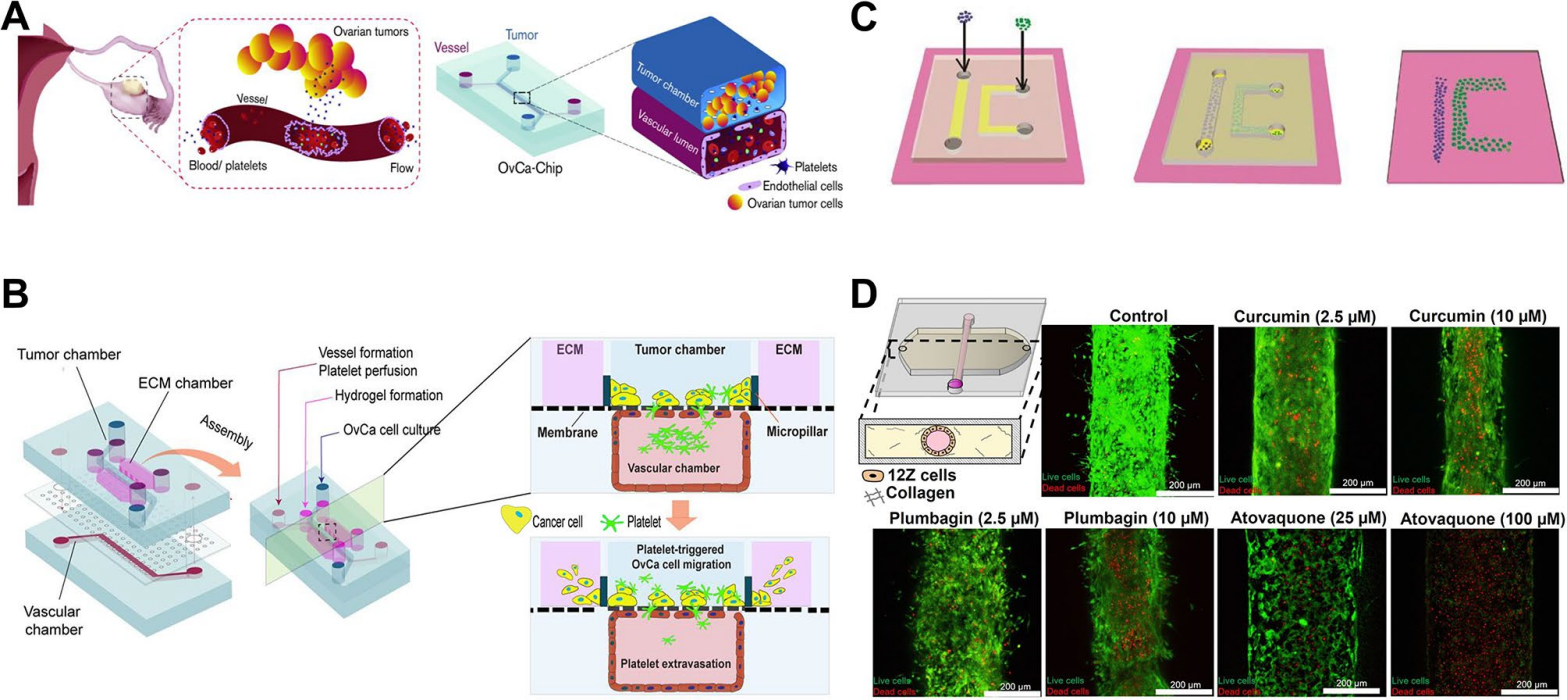
Mimicking cancer and endometriosis on the chip. (**A**) Organ-on-a-chip model of vascular-platelet crosstalk in ovarian cancer. Damaged blood vessels in ovarian cancer are adjacent to the tumor, and the two fluidic chambers in the chip (red: blood vessel chambers; blue: cancer cell chambers) are also in adjacent superimposed positions. Reproduced with permission [19]. Copyright 2020 The American Society of Hematology. (**B**) Engineering drawing of the microdevice containing two PDMS compartments separated by a thin porous membrane that reproduces the microarchitecture of the tumor-vascular interface (left). On the right, cross-sectional side view of the ovarian tumor microenvironment chip describes tissue organization inside the chip. Reproduced with permission [128]. (**C**) Model for simulating the interaction between endometrial stromal cells (ESCs) and human peritoneal mesothelial cells (HPMCs). Cells were inoculated in microchannels, and the straight microchannel was inoculated with ESCs (blue), and the U-shape microchannel was inoculated with HPMCs (green). Reproduced with permission [14]. Copyright 2012 The Royal Society of Chemistry. (**D**) Schematic of the microfluidic device used to study the proliferation and metabolic status of endometriotic 12Z cells. Green dye labelled live cells and red dye labelled dead cells. The device was used to test the effects of curcumin, plumbagin and atovaquone on 12Z cell viability. Reproduced with permission [137]. Copyright Society for Reproduction and Fertility 2023

Ovarian cancer is the most common malignant tumor of the female reproductive system and can be classified into various histological subtypes, with epithelial ovarian tumors accounting for 90% of all ovarian tumors [123]. Traditional ovarian cancer research mostly relies on 2D cell and animal models, but their ability to extrapolate experimental data to predict in vivo responses is limited [124]. With the advancement of bio-fabrication and reactor technology, a 96-well microplate bioreactor platform was developed to expose constructed ovarian cancer models to anticancer drugs, demonstrating the utility of dual perfusion bioreactor platforms for throughput and drug screening [125]. Later, microfluidic chips capable of isolating ovarian cancer exosomes and establishing their protein profiles were also developed [126, 127]. As the knowledge of the tumor microenvironment deepens, more and more models are being used to elucidate the interactions between blood vessels, tumor cells, and fibroblasts within ovarian cancer. *Saha et al.* co-cultured A2780 human ovarian cancer cells with HUVECs to establish the first ovarian cancer-on-a-chip that mimics the cancer-vascular-hematologic relationships, demonstrating the potential of this system as a preclinical drug testing platform [19] (Fig. 9A). However, to enable longitudinal studies of cancer progression and to analyze the effect of extravasated platelets on cancer cell proliferation and invasiveness, their team redesigned the top tumor compartment of the device by adding an extracellular matrix compartment on each side, separated by an array of polydimethylsiloxane (PDMS) micro-posts, to develop an ovarian tumor microenvironment chip (OTME-Chip) (Fig. 9B). They inoculated human ovarian microvascular endothelial cells (HOMECs) in the lower chamber of the device to form a complete 3D vascular lumen [128]. They used the OTME-Chip in combination with gene editing and next-generation RNA sequencing tools to advance the discovery of novel antiplatelet therapies targeting tumor metastasis and chemotherapy resistance. Recently, *Fedi et al.* implemented a co-culture model by culturing ovarian cancer SKOV3 cells in 24-well plates housing 24-well transwell inserts in which the liver HepG2 cell line was spread on the bottom. They then applied a closed-loop fluid simulating a drug propagation circulatory system via a pumping system, and developed an abbreviated multicompartmental OOC model fluidically connecting 3D ovarian cancer tissues to hepatocytes for simultaneous study of chemotherapeutic drug efficacy and hepatotoxic effects in a physiological setting [129]. Additionally, the fallopian tube origin of ovarian cancer has been reported [130], and *Ferraz et al.* used canine fallopian tube tissue to create a the fallopian tube-on-a-chip model to facilitate the understanding of the tubal origin of ovarian cancer by modelling the transformation of the fallopian tube epithelium [131].

Besides ovarian cancer, *Kim’s* group developed a multichannel cell chip containing a 3D scaffold that incorporates not only human glioblastoma, normal liver cells, and normal lung cells, but also cervical cancer cells, allowing simultaneous drug screening in multiple cells. This has been shown to be a new method for cancer treatment and may also be an excellent tool for analyzing the biological process of cervical cancer [132].

These models may help to deepen the understanding of cancer staging, identify effective biomarkers of early and metastatic cancer, and develop new targeted therapies in the future.

### **OOC model of other disease**

#### **Endometriosis (**Table 3, **lines 12–13)**

Endometriosis is a disorder characterized by the extrauterine ectopic growth of endometrial epithelium and stroma, which can cause severe pelvic pain and impair fertility [133]. Currently, there is no known cure for endometriosis, and the treatment is usually aimed at symptom management. Most human endometriosis studies rely on animal models, however, the lack of spontaneous endometriosis development in rodents poses a great challenge to understanding the pathogenesis of this disease [134]. Some researchers have summarized the challenges of modeling the pathophysiology of endometriosis with unknown etiology and suggested that the emerging microfluidic organ chip technology could be used to model the endometrium in vitro and better elucidate the pathophysiological features of the disease [135]. In 2012, *Chen et al.* developed the first microfluidic in vitro model of endometriosis, which used microfluidic channels with coverslips to mimic the pathology of peritoneal endometriosis and enabled the patterning and release of endometrial stromal cells (ESCs) and human peritoneal mesothelial cells (HPMCs) [14] (Fig. 9C). They found that HPMCs from both control and endometriotic groups could resist the invasion by ESCs, whereas HPMCs from endometriotic individuals could not resist the invasion by ESCs from either normal and endometriotic individuals. A subsequent study employed a microfluidic system to investigate the determination of multiple protease activities in endometriosis patients [136]. Recently, *Kapur et al.* reported an OOC lumen model of endometriosis, in which 12Z cells (an endometriosis cell line) were inoculated into the 3D lumen of microdevices suspended in a collagen hydrogel, and atovaquone, plumbagin, or curcumin were added to the lumen to assess their effects on cell viability [137] (Fig. 9D). They screened for the potential repurposing of atovaquone, an anti-malarial drug, for the effective treatment of endometriosis.

Although several OOC microphysical systems have been developed in recent years to directly study the uterine stromal vascular remodeling, hormonal changes, and organ-to-organ reciprocal endocrine crosstalk during the menstrual cycle (as previously described) [17, 138], the field of organ microarrays of the ectopic endometrium remains in dire need of development to unravel the complex and multifactorial pathogenic processes.

#### Preeclampsia

Preeclampsia (PE) is a hypertensive pregnancy complication that may lead to insufficient blood supply to the placenta, reduce fetal access to oxygen and nutrition, fetal distress, and even stillbirth [139]. Although previous placental barrier chip models have been mentioned to be applicable in diseases such as preeclampsia, which is associated with uteroplacental hypoxia [64, 94], very few OOC models have directly simulated the pathophysiology of preeclampsia. *Rabussier’s* team was the only one to construct a placenta-on-a-chip with syncytial differentiation, barrier function, hormone secretion, and transport capabilities [94] (Table 2, **row 7**). To mimic the features related to placental hypoxic pathology in the placental barrier chip model, they exposed the model to hypoxia and ischemia and found that the placenta exhibited PE-like characteristics, such as impaired syncytial differentiation and barrier function, and downregulated expression of transporters and angiogenic factors [140–142]. This is the first placental barrier-on-a-chip to simulate the pathological features related to uteroplacental hypoxia and may improve our understanding of the pathogenesis of preeclampsia.

## Current limitations of the OOC platform

Despite the successful application of OOC technology to numerous human organs, its real-world applicability remains hindered by a multitude of natural and technical challenges:

1) Cell source constraints: A prevalent challenge encountered by OOC developers and users alike is the procurement of renewable cells. This primarily stems from the inability of most OOC platforms to proliferate primary cells, necessitating the establishment of cell cultures directly from donors or patients. The selection of suitable cellular components for the system is contingent upon the platform’s operational environment and the availability of specific cell sources from commercial providers or primary donors.

2) Cell culture environment constraints: This encompasses factors such as the type of culture medium, temperature, oxygen concentration, and fluid flow rate within the organ chip environment. Our literature review also prioritized the extraction of this information, revealing that even OOC platforms developed by the same team, using co-cultures of identical cell types, employed varying medium ratios. The type of medium undeniably exerts a direct influence on the cellular state, necessitating more systematic future studies on aspects such as medium composition, fluid flow rate, and shear stress.

3) Material selection challenges for OOC: A microfluidic chip is composed of a series of grooves or microchannels etched onto various materials, including glass, silicon, polymethylmethacrylate (PMMA), and polydimethylsiloxane (PDMS) [143]. PMMA, a transparent plastic material, boasts advantages such as excellent optical properties, low cost, and ease of processing. However, it also suffers from drawbacks such as inferior mechanical properties, susceptibility to aging, and protein adsorption [144]. Glass and silicon, traditional materials for nanofabrication, offer superior biocompatibility, high processing precision, and are suitable for standard photolithography and etching processes. Yet, they require specialized processing equipment, and silicon’s poor light transmission is not conducive to observing tissues within the chip [145]. Consequently, PDMS has emerged as the preferred material for most pioneering work due to its non-toxicity, biocompatibility, low cost, good light transmittance, and air permeability. It remains one of the most extensively used materials in device fabrication [146], as evidenced in our summarized table. However, PDMS does have certain drawbacks [147, 148]. For instance, PDMS absorbs biological and pharmaceutical hydrophobic small molecules from solution, which can impact the accuracy of drug screening, and it adsorbs proteins on its surface. Moreover, leakage of unpolymerized monomers in PDMS may cause cellular toxicity. These drawbacks could potentially bias the final results. In the future, if the use of PDMS cannot be circumvented, its surface properties could be enhanced by covalently immobilizing various molecules.

4) Challenges in selecting germination growth substrates for OOC: The OOC is a micro-biological system that replicates the functions of human organs. It necessitates the selection of appropriate materials to be placed on the device substrate, which acts as a germination and growth matrix to facilitate cell growth and differentiation. Collagen and hydrogel are two commonly utilized substrate materials. Collagen, a naturally occurring protein extensively found in the human body’s extracellular matrix, exhibits good biocompatibility and biodegradability, promoting cell adhesion, migration, and extracellular matrix formation [149]. However, collagen’s complex preparation process, batch variation, low mechanical strength, and susceptibility to microbial contamination can impact the stability and reproducibility of organ chips. Hydrogel, a highly hydrophilic and optically transparent polymer with high water content, can serve as a germination and growth substrate, providing cells with nutrients and oxygen while mimicking the physical and chemical properties of extracellular matrices. Hydrogels are relatively simple to prepare, and their mechanical strength and porosity can be adjusted to accommodate different cell types and functions by altering the type and concentration of cross-linking agents. However, hydrogels are less biocompatible and stable, are easily degraded by enzymes secreted by cells, and lack specific adhesion molecules on their surface, necessitating surface modification to enhance cell adhesion and differentiation [150]. Both collagen membranes and hydrogels have their respective advantages and disadvantages as matrix materials for organ chips, making it essential to select suitable materials or combine them to achieve optimal results based on different organs and application purposes.

5) Lack of universal criteria for determining modeling success: How does one ascertain the success of a constructed OOC? For instance, in the case of placenta-on-a-chip, some researchers currently undertake analyses of placental barrier permeability and glucose transfer to characterize the success of the modelling. However, the field of organ chips lacks universal standard regulations for determining modelling success, which has had adverse effects on organ chip research and the market. These include: (i) leading to unclear definitions and classifications of organ chips, resulting in conceptual confusion and misinformation, e.g., organ-like, organ chips, and microphysiological systems. (ii) limiting the quality and reliability of organ chips, thereby affecting their biological relevance and predictive properties. (iii) impeding the communication and cooperation of organoids, and reducing the sharing and compatibility of organoids, e.g., data, platforms, and interfaces of organoids. (iv) increasing the risk and uncertainty of organoids, and constraining their recognition and application, e.g., ethical, safety, and efficacy of organoids. Therefore, the establishment of common standards and regulations for determining the success of modelling is an urgent need in the field of OOC. It is an important guarantee for the development of OOC and will further regulate the research and market of OOC.

6) Challenges in simulating the in vivo environment: The female reproductive system is a highly intricate and dynamic biological system that involves the interaction and regulation of multiple cell types, cytokines, and endocrine hormones. Precisely designing and integrating the complex crosstalk between cytokines and endocrine hormones that exist in the female reproductive system, within a microliter volume of fluid and a scaled-down tissue and organ structure, remains a formidable challenge. Another hurdle to overcome is maintaining proper vascularization and cell-cell interactions to provide adequate nutrients and oxygen, as well as to mimic signaling under physiological and pathological conditions.

## Conclusion and prospects

In recent decades, tremendous advances have been made in our basic understanding of human reproductive systems. However, the laboratory-level simulation of the pathophysiological state of female reproductive systems remains largely underdeveloped. Organ-on-a-chip (OOC) technology, an interdisciplinary field that integrates material fabrication, microfluidics, life sciences, and 3D bioprinting [151], offers a powerful approach to reconstruct the complexity of the human reproductive system and its physiological and pathophysiological processes. This review summarizes the latest progress in the application of OOC in the pathophysiological models of the female reproductive system. It focuses on its capability and potential in simulating the function and physiological state of various organs and tissues of the female reproductive systems, as well as its application in the field of reproductive science, particularly in placental modeling. It also reviews and synthesizes its application in the pathophysiology modeling of reproductive diseases. We believe OOC technology will be the future of pathophysiological modelling of the female reproductive system.

OOC technology can bring more opportunities to the field of medical science in the future, including:

1) Integration of mass spectrometry for early cancer detection: Mass spectrometry, a method for analyzing the mass and structure of a substance, can be employed to detect metabolites, proteins, peptides and other molecules in biological samples. By integrating OOC technology with mass spectrometry, it becomes feasible to simulate the metabolism and secretion of cancer cells, as well as the interaction between cancerous and normal tissues. This enables the discovery of cancer biomarkers, thereby facilitating early diagnosis and prognosis evaluation of cancer.

2) Toxicological studies of the female reproductive system, drug transport and drug efficacy assessment: OOC models of the female reproductive system can simulate the absorption, distribution, metabolism and excretion of drugs or other substances in the reproductive system, as well as their effects on reproductive functions. This allows for the assessment of the safety and efficacy of drugs, as well as the detrimental effects of toxins and pollutants. These models can be used for pharmacokinetic and pharmacodynamic analyses, and for testing the impacts of drugs, toxins, and environmental pollutants on reproductive function.

3) Integration with high-throughput sequencing tools for diseases mechanisms studies: High-throughput sequencing, a rapid, efficient, and cost-effective method for determining DNA or RNA sequences, can be utilized to study gene expression, mutation, regulation, and other molecular-level information. By integrating OOC technology with high-throughput sequencing tools, it becomes feasible to simulate the onset and progression of diseases and the mechanism of drug action. This approach can reveal the molecular mechanisms of diseases and facilitate the discovery of new drug targets and therapeutic strategies.

4) Development of automated high-throughput systems and biosensors: An automated high-throughput system employs robots, sensors, computers, and other equipment to achieve large-scale, rapid, and accurate experimental operations and data analysis, thereby enhancing the efficiency and reliability of OOC technology. Biosensor devices, which use biological molecules or cells as recognition elements to convert biological signals into detectable electrical signals, can be employed for real-time, continuous, and non-invasive monitoring of physiological parameters, such as pH, temperature, oxygen, and pressure in OOC techniques. Through the development of automated high-throughput systems and biosensors, OOC technology can be rendered intelligent and precise, thereby expanding the scope and value of its applications.

What should be the focal points in future OOC-based research?

1) Enhancing the complexity of OOC models: Currently, many OOC models primarily concentrate on the function of a single organ. However, the organs of the human body are highly interactive and function in unison, and research focusing on a single organ cannot fully encapsulate the complexity of the human body. In the process of augmenting the complexity of OOC models, two aspects need to be considered. Firstly, the biological complexity of the model, which includes incorporating multi-organ systems, introducing immune cells into the model to simulate the body’s immune response, and constructing more complex vascular networks and tissue structures. Secondly, the realization of biological complexity depends on the enhancement of technical complexity, such as dynamic 3D culture, controllable physicochemical microenvironment, organ-like vascularization, biosensing, and the effective integration of other advanced technologies (such as gene editing, biomaterials, artificial intelligence, etc.). Increasing the complexity of OOC models aids in improving the physiological relevance of the model, enabling OOC models to play a more significant role in biomedical research and drug development. In the future, it is anticipated that there will be a trend towards developing more complex OOC models that simulate the interaction and communication between multiple organs.

2) Addressing the challenges of long-term dynamic studies in OOC: The application of actuators offers a potential solution. Actuators can simulate physiological mechanical stimuli in the body, such as blood flow and respiratory movements, bringing the OOC models closer to the actual physiological environment. However, the technical challenges of effectively integrating actuators with OOC models and controlling the actuators to generate precise physiological mechanical stimuli need to be addressed [152]. Furthermore, the development of new sensors and detection technologies can not only aid in controlling the operation of actuators more accurately but also assist in monitoring the changes in cells/tissues during the long-term culture process in real-time. This can lead to the development of long-term culture OOC models that are comparable to animal models.

3) Addressing the standardization and repeatability issues of various OOC platforms: The design and methods of organ chips differ among researchers. Therefore, increased efforts towards the standardization of OOC design techniques, result verification, quality control, and open sharing would be beneficial. (i) Technical standardization: Establish unified technical specifications for the production and operation of organ chips. This includes standards for the design, production, cell planting, and culture conditions of organ chips, to ensure the consistency of organ chips produced and operated by various laboratories. (ii) Data standardization: Establish unified standards for data collection and analysis, including methods for data collection, data processing, and analysis. This ensures that data obtained from different laboratories can be effectively compared and integrated. (iii) Result verification: Through multi-center research, verify the production and operation results of the same organ chip in different laboratories to ensure the repeatability of its results. (iv) Quality control: Establish a strict quality control system to monitor the entire process of the production and operation of organ chips to ensure the stability of its quality. (v) Open sharing: Establish an open sharing platform, such as the Organ Chip Database (OCDB, http://www.organchip.cn/?lang=en_US), to share the design, production, and operation technical information of organ chips, as well as related research data and results, to promote the exchange and sharing of information.

These measures can contribute to resolving the standardization and repeatability issues associated with the organ chip platform, thereby fostering the development and application of organ chip technology. However, the successful implementation of these measures necessitates the collaborative efforts and cooperation of various research institutions, enterprises, and regulatory agencies.

4) Promoting wider acceptance and application of OOC technology in clinical research: Despite being developed for 15 years, the application of OOC technology in clinical settings remains limited. The reasons may include: (i) Insufficient maturity: While OOC technology has evolved over time, it may not be fully mature and may require further validation and improvement. (ii) Limited model complexity: Current technology may not yet be capable of constructing immune microenvironment models, reproducing vascularization processes, or achieving multi-organ interactions. This limits the evaluation of large molecule drugs that rely on antibody-dependent cell-mediated cytotoxicity (ADCC), immunosuppressive drugs, and anti-angiogenic drugs. (iii) It is currently challenging to standardize OOC culture systems, resulting in poor reproducibility and stability, which cannot support large-scale and automated applications. (iv) Cost concerns: Due to the precision and technical nature of their operation, the cost of organ-on-chip devices is unlikely to decrease significantly. (v) Limitations of non-clinical research: The limitations of non-clinical OOC models, which cannot accurately predict the effects of drugs in the human body 100% of the time, may lead to many non-clinical studies failing when transitioning to clinical research.

To gain broader acceptance in clinical practice, OOC models not only need to improve the maturity of the technology but also need to be validated against clinical outcomes and incorporated into the drug approval regulatory framework. In addition, strengthening collaboration among biomedical engineers, clinical doctors, and drug developers is essential to ensure that OOC technology meets clinical needs and addresses real-world problems. Incorporating knowledge of OOC technology into medical education and professional training to enhance clinicians’ awareness and ability to use the technology is crucial for the promotion of OOC technology in clinical research.

## Data Availability

Not applicable.
